# Metal-Organic Frameworks Offering Tunable Binary Active Sites toward Highly Efficient Urea Oxidation Electrolysis

**DOI:** 10.34133/2022/9837109

**Published:** 2022-06-27

**Authors:** Xuefei Xu, Qingming Deng, Hsiao-Chien Chen, Muhammad Humayun, Delong Duan, Xia Zhang, Huachuan Sun, Xiang Ao, Xinying Xue, Anton Nikiforov, Kaifu Huo, Chundong Wang, Yujie Xiong

**Affiliations:** ^1^School of Optical and Electronic Information, Wuhan National Laboratory for Optoelectronics, Optics Valley Laboratory, Huazhong University of Science and Technology, Wuhan 430074, China; ^2^Physics Department and Jiangsu Key Laboratory for Chemistry of Low-Dimensional Materials, Huaiyin Normal University, Huaian 223300, China; ^3^Center for Reliability Science and Technologies, Chang Gung University, Taoyuan, 33302 Taiwan, China; ^4^School of Chemistry and Materials Science, University of Science and Technology of China, Hefei, 230026 Anhui, China; ^5^Department of Physics, College of Science, Shihezi University, Xinjiang 832003, China; ^6^Department of Applied Physics, Ghent University, Gent 9000, Belgium

## Abstract

Electrocatalytic urea oxidation reaction (UOR) is regarded as an effective yet challenging approach for the degradation of urea in wastewater into harmless N_2_ and CO_2_. To overcome the sluggish kinetics, catalytically active sites should be rationally designed to maneuver the multiple key steps of intermediate adsorption and desorption. Herein, we demonstrate that metal-organic frameworks (MOFs) can provide an ideal platform for tailoring binary active sites to facilitate the rate-determining steps, achieving remarkable electrocatalytic activity toward UOR. Specifically, the MOF (namely, NiMn_0.14_-BDC) based on Ni/Mn sites and terephthalic acid (BDC) ligands exhibits a low voltage of 1.317 V to deliver a current density of 10 mA cm^−2^. As a result, a high turnover frequency (TOF) of 0.15 s^−1^ is achieved at a voltage of 1.4 V, which enables a urea degradation rate of 81.87% in 0.33 M urea solution. The combination of experimental characterization with theoretical calculation reveals that the Ni and Mn sites play synergistic roles in maneuvering the evolution of urea molecules and key reaction intermediates during the UOR, while the binary Ni/Mn sites in MOF offer the tunability for electronic structure and *d*-band center impacting on the intermediate evolution. This work provides important insights into active site design by leveraging MOF platform and represents a solid step toward highly efficient UOR with MOF-based electrocatalysts.

## 1. Introduction

As urea (CO(NH_2_)_2_) widely exists in industrial effluents and domestic sewage, urea-rich wastewater has become the main source of water pollution in recent years due to eutrophication, leading to the damage of ecological environment. Specifically, it could be transformed into nitrate and toxic ammonia, posing a threat to the human's health [1, 2]. For this reason, the electrooxidation treatment of urea in wastewater that can convert the urea into harmless N_2_ and CO_2_ has received great attention from the research community [3, 4]. Moreover, such a urea oxidation reaction (UOR) is also the underlying half reaction that can be coupled with hydrogen production [5], urea fuel cells [3], and artificial kidneys [6], directly determining their efficiency. Nevertheless, limited by the complex six-electron transfer process and multiple intermediate adsorption/desorption steps, the UOR generally suffers from intrinsically sluggish kinetics [7].

To overcome the limitation, it is imperative to develop highly efficient electrocatalysts, for which the rational design of catalytically active sites holds the key. It has been recognized that among various materials including precious metal-based compounds, nickel (Ni)-based catalysts exhibit higher oxidation current densities for UOR in alkaline media where the Ni^3+^ species acts as the catalytically active sites for urea oxidation [8, 9]. To this end, great efforts have been devoted to optimize the ratio of Ni^3+^/Ni^2+^ in Ni-based catalysts through adjusting morphological structures [10–12], decorating surfaces [13, 14], and doping heterogeneous elements [15]. Nevertheless, it remains challenging to maneuver the adsorption and evolution of multiple reaction intermediates on a single type of catalytic sites due to the inherent scaling relationship in the oxygen-related electrocatalytic process [16, 17], limiting the overall catalytic performance. Most recently, Zhang et al. have reported that the incorporation of a second adsorption site into a matrix can allow various intermediates adsorbed in the two different sites, which in turn favors to break the scaling relationship [16]. Specifically, CO(NH_2_)_2_∗ is adsorbed at the Co site and donates protons to the OH∗ species anchored at the Mo site, forming short hydrogen bonds (Mo–HO∙∙∙H–NHCONH_2_–Co), which facilitates the breakage of N–H bonds. Wang et al. have also demonstrated the synergistic roles of Co and Mn sites in UOR using CoMn-layered double hydroxide (LDH) catalyst [18]. As such, it is anticipated that the design of binary active sites in Ni-based catalysts should be a promising approach to achieve the advanced UOR performance.

In the commonly used inorganic materials, however, their rigid crystalline structures largely limit the tunability of binary active sites. Furthermore, key fundamental issues concerning the active sites (e.g., genuine origin of intermediates and catalytic activity) have not been elucidated so far, which calls for determination of the active intermediate species at the atomic level during the UOR process but is hindered by the complexity of catalyst surface structures. Metal-organic frameworks (MOFs), constructed with well-dispersed metal nodes and organic linkers, have been attempted as electrocatalysts due to the advantages of their periodic porous structures, numerous exposed active sites, and large specific surface areas [19–21]. The tunable metal nodes enable the incorporation of multiple active sites into a MOF, while the well-dispersed metal sites and homogeneous microenvironment of MOFs facilitate to understand the synergistic effect of the active sites [22–24]. As such, MOFs offer an inherent advantage for both mechanism studies and performance optimization. Nevertheless, the design of multiple active sites in MOFs for UOR process remains largely unexplored.

In this work, we have synthesized bimetallic MOF, NiMn-BDC based on Ni/Mn sites and terephthalic acid (BDC) ligands, through a one-pot solvothermal process. The optimal NiMn_0.14_-BDC catalyst exhibits a low voltage of 1.317 V to deliver a current density of 10 mA cm^−2^, which outperforms monometallic Ni- and Mn-MOFs. Moreover, the urea degradation over the NiMn_0.14_-BDC supported on nickel foam (NiMn_0.14_-BDC/NF) reaches 97.65%, 96.71%, and 81.87% in 0.0033 M, 0.033 M, and 0.33 M urea solution, respectively, demonstrating that the catalyst possesses excellent potential for wastewater treatment. Such a UOR performance highly relies on the atomic ratio of Mn/Ni sites. Our systematic investigations reveal that the binary Mn/Ni sites offer tunable electronic structures and *d*-band centers, and provide synergistic sites for maneuvering the adsorption and activation of urea molecules as well as the evolution of key NH∗ and CO∗ intermediates, which thereby reduces the energy requirements for UOR. The synergistic effect of the Ni and Mn sites demonstrated in NiMn-BDC here should be insightful for designing multiple active sites toward high-performance UOR electrocatalysis.

## 2. Results

### 2.1. Catalyst Design, Synthesis, and Characterization

**Figure 1:**
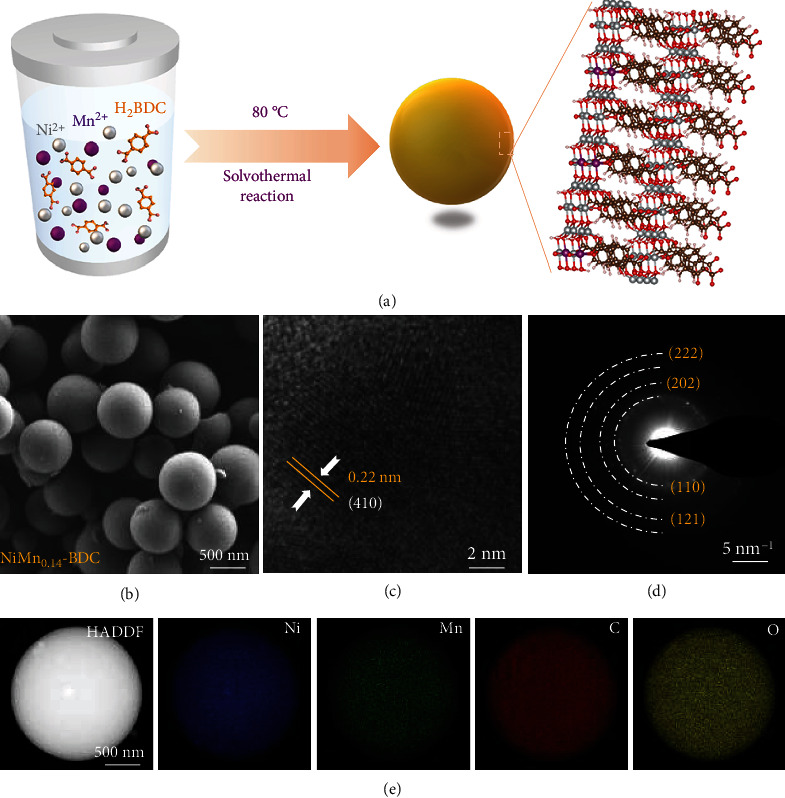
Schematic synthetic process and morphology characterization. (a) Schematic illustration for the synthesis of NiMn-BDC. (b) TEM image, (c) HRTEM image, (d) SAED pattern, and (e) TEM-EDS elemental mapping of the Ni, Mn, C, and O elements of NiMn_0.14_-BDC.

The NiMn-BDC MOFs are synthesized via a one-pot solvothermal process by using metal nitrates (Ni(NO_3_)_2_·6H_2_O and Mn(NO_3_)_2_·4H_2_O) as metal sources and terephthalic acid (H_2_BDC) as organic linkers. The schematic synthetic process of NiMn-BDC is shown in Figure 1(a). For comparison, a series of NiMn-BDC MOFs with different ratios of Mn/Ni are prepared by tailoring the amount of Mn(NO_3_)_2_·4H_2_O precursor. The contents of Ni and Mn are determined by inductively coupled plasma-optical emission spectrometer (ICP-OES) (Figure S1). For simplicity, the as-prepared samples are termed as NiMn*_x_*-BDC, in which *x* is the molar ratio of Mn to Ni. The photographs in Figure S2 show that the as-prepared NiMn*_x_*-BDC are green powders. According to scanning electron microscopy (SEM) images, all the as-prepared NiMn*_x_*-BDC MOFs exhibit spherical structures with a diameter of ~800 nm) (Figure 1(b), S3), suggesting that the incorporation of Mn has not affected the morphology of Ni-BDC. In contrast, the monometallic Mn MOF (Mn-BDC) exhibits a micron-sized bulk structure without specific morphology. To gain more structural information, high-resolution transmission electron microscopy (HRTEM) has been employed to examine the Ni-BDC and NiMn_0.14_-BDC. As shown in Figure 1(c), the fringe spacing of NiMn_0.14_-BDC is 0.22 nm, indexed to the (410) plane of MOF, which is very similar to that of Ni-BDC (Figure S4) [25]. The polycrystalline nature of our NiMn_0.14_-BDC is evidenced by selected-area electron diffraction SAED patterns (Figure 1(d)). To resolve elemental distribution, we further record energy-dispersive X-ray spectroscopy (EDS) mapping (Figure 1(e)), revealing that C, O, Ni, and Mn elements are evenly distributed over the entire sphere of NiMn_0.14_-BDC. Taken together, these observations suggest that the Mn element has been successfully incorporated into the parent Ni-BDC.

**Figure 2:**
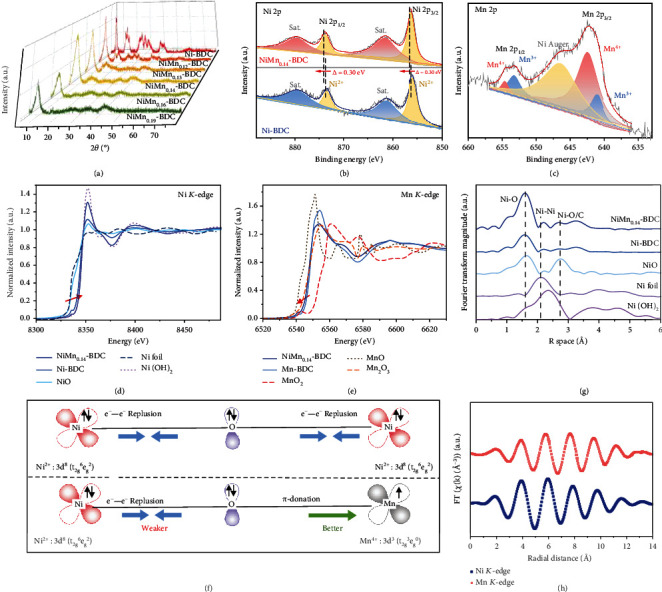
Structural characterization. (a) XRD patterns of the as-prepared NiMn*_x_*-BDC MOFs. (b) High-resolution XPS spectra of Ni 2p for Ni-BDC and NiMn_0.14_-BDC. (c) XPS core-level spectra of Mn 2p for NiMn_0.14_-BDC. XANES spectra of Ni-BDC, NiMn_0.14_-BDC, and reference materials at the (d) Ni *K*-edge and (e) Mn *K*-edge. (f) Schematic illustration of the electronic coupling among Ni, O, and Mn in Ni-BDC and NiMn_0.14_-BDC. (g) EXAFS spectra of Ni *K*-edge for Ni-BDC, NiMn_0.14_-BDC, and reference materials. (h) EXAFS spectra (*K* space) of Ni *K*-edge and Mn *K*-edge for NiMn_0.14_-BDC.

Figure 2(a) shows the X-ray diffraction (XRD) patterns of our samples. The XRD peaks of Ni-BDC are well indexed to the MOF of Ni-BDC (Ni_2_(OH)_2_(C_8_H_4_O_4_)) (Figure S5a) [26, 27]. After careful scrutinizing, we recognize that some of the characteristic peaks become weaker or even disappear with the increase of Mn content in NiMn-BDC, suggesting that the incorporation of Mn has reduced the long-range order of the parent Ni-BDC. This crystallinity change is most likely related to the fact that the doped high-charged Mn triggers the new electron distribution around Ni center, resulting in the aperiodic arrangements of atoms [28]. Previous research via density functional theory (DFT) calculation has indicated that the defect formation energies of bimetallic MOF (e.g., CoFe-MOF) can be dramatically reduced by increasing the content of second metal nodes (Fe) [29]. Since the Fourier transform infrared (FT-IR) spectra of Ni-BDC and NiMn_0.14_-BDC are nearly identical (Figure S5b), it is believed that the NiMn_0.14_-BDC should still retain the crystal structure of parent Ni-BDC after Mn nodes are introduced. In our collected EPR spectra (Figure S6), a more pounced symmetric peak at *g* = 2.002 is observed for the NiMn_0.14_-BDC sample in comparison with that of the Ni-BDC, faithfully validating that more oxygen vacancies are created in NiMn_0.14_-BDC due to the Mn incorporation [30–32]. Given that the metal sites with unsaturated coordination are usually catalytically active centers [11], the numerous defects induced by Mn incorporation will allow the metal sites exposed to reaction species, which in turn leads to the enhanced catalytic activity of the NiMn-BDC. It turns out that the specific surface area of the NiMn_0.14_-BDC (268.55 m^2^g^−1^) is nearly twice that of the Ni-BDC (136.61 m^2^g^−1^) (Figure S7).

To examine the chemical and electronic states of NiMn_0.14_-BDC, X-ray photoelectron spectroscopy (XPS) characterization is performed. As expected, C, O, Mn, and Ni elements can be clearly differentiated in the XPS survey spectrum of NiMn_0.14_-BDC (Figure S8(a)), in line with the EDS elemental mapping. As a reference, the Ni 2p spectra of Ni-BDC (Figure 2(b)) reveal two distinct peaks at 856.1 and 873.7 eV, which correspond to the Ni 2p_3/2_ and Ni 2p_1/2_ orbitals of Ni^2+^, respectively [33]. In comparison, the Ni 2p peak of NiMn_0.14_-BDC is slightly shifted toward higher binding energy, suggesting the electron loss from Ni [34]. The O 1s spectra of NiMn_0.14_-BDC in Figure S8b can be deconvoluted into three peaks. The peaks centered at 531.5, 532.4, and 533.8 eV are assigned to the O-Ni/Mn, O-C=O, and adsorbed water species, respectively [27]. In the Mn 2p spectra of NiMn0.14-BDC (Figure 2(c)), two deconvoluted peaks centered at 641.1 and 642.5 eV indicate the existence of Mn^3+^ and Mn^4+^ species, respectively [35]. Overall, the XPS characterization reveals that the involvement of Mn alters the local electronic states of the Ni-based MOF.

To resolve the local information for NiMn-BDC, X-ray absorption fine structure (XAFS) spectroscopy is employed to characterize the Ni-BDC and NiMn_0.14_-BDC. As displayed in Figure 2(d), the overall X-ray absorption near-edge structure (XANES) profile of NiMn_0.14_-BDC is similar to that of the Ni(OH)_2_, with a weak pre-edge peak at ca. 8333.0 eV, suggesting that the Ni atoms in NiMn_0.14_-BDC have +2 oxidation state and are coordinated octahedrally [36]. As compared with the case of Ni-BDC, positive shift can be observed in the near-edge adsorption region of NiMn_0.14_-BDC, signifying that the valence of Ni in NiMn_0.14_-BDC is increased after the introduction of Mn, in consistent with the XPS result [37]. Moreover, the Mn *K*-edge XANES spectra are also recorded as shown in Figure 2(e). The *K*-edge of Mn in NiMn_0.14_-BDC situates between those of the Mn_2_O_3_ and MnO_2_, suggesting that the valance state of Mn is between +3 and +4, in line with the XPS observation as well. In comparison with the Mn-BDC, a slightly positive shift is discerned in the Mn *K*-edge XANES spectra of NiMn_0.14_-BDC, further validating the fact that the electrons are transferred from Ni to Mn. From another perspective, the *π*-symmetry (t_2g_) *d*-orbitals of Ni^2+^ are fully occupied, which induces a strong e^−^–e^−^ repulsion between the bridging O^2−^ and Ni^2+^. In contrast, Mn^4+^ possesses three unpaired electrons in the *π*-symmetry (t_2g_) *d*-orbital, interplaying with the bridging O^2−^ via *π*-donation [38, 39]. In the NiMn_0.14_-BDC, there are few occupied electrons in the t_2g_ orbital of Mn^4+^ in comparison with that of the Ni^2+^, which allows to accommodate the electrons in the t_2g_ orbital of Mn^4+^. This feature essentially induces the electron transfer from Ni to the adjacent Mn via O^2-^ bridging. The configured Ni-O-Mn model for displaying the electronic interplay between Ni and Mn is shown in Figure 2(f).

To look into bonding information, extended XAFS (EXAFS) spectra are analyzed. The EXAFS spectra exhibit an obvious Ni peak at roughly 1.60 Å and a Mn peak at roughly 1.62 Å (Figure 2(g) and Figure S9), which are ascribed to the isolated Ni(or Mn)-O first coordination shell. Furthermore, another minor peak at 2.86 Å (or 2.64 Å) is associated with the Ni(or Mn)-C(or O) second coordination shell, confirming that both Ni and Mn species are in their single-site forms in NiMn_0.14_-BDC [27, 36]. It should be noted that Mn and Ni *K*-edge appear very similar in shape and oscillating frequency (Figure 2(h)), suggesting that the coordination situation of Ni and Mn nodes in NiMn_0.14_-BDC is quite comparable. According to EXAFS fitting results (Figure S10 and Table S1), the best fitting for both the Ni and Mn coordination spheres is the coordination with six oxygen atoms. As such, the Mn atoms should have replaced the Ni atoms in the as-prepared NiMn_0.14_-BDC. Given the same coordination number of Ni-O path in Ni-BDC and NiMn_0.14_-BDC, we can conclude that the main framework of Ni-BDC is well maintained after Mn incorporation.

### 2.2. Electrocatalytic UOR Performance

**Figure 3:**
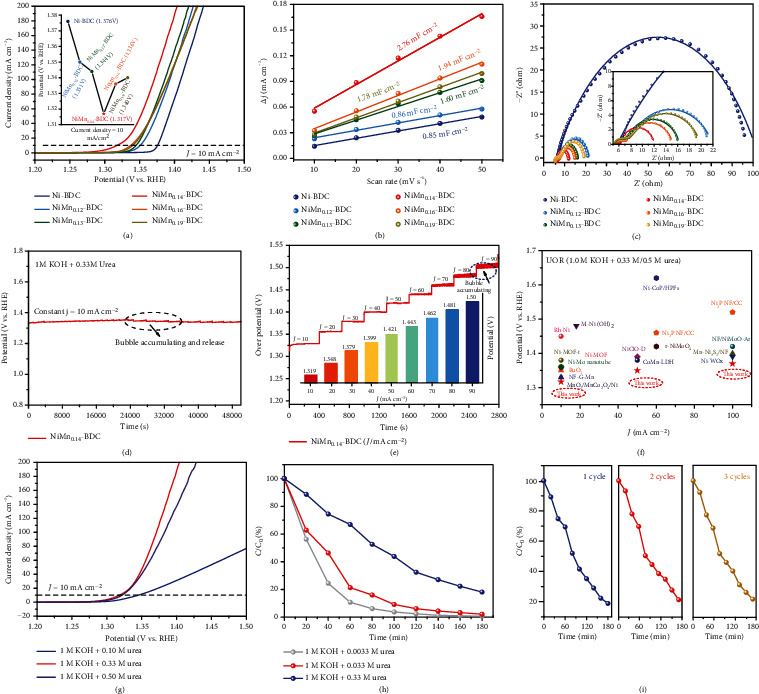
Electrocatalytic UOR measurements. (a) 95%-iR corrected LSV curves of the samples measured in 1 M KOH electrolyte with 0.33 M urea. The insert shows the relationship between Mn/Ni ratio and UOR overpotential at 10 mA cm^−2^. (b) *C*_dl_ of NiMn*_x_*-BDC derived from current density versus scan rate. (c) Nyquist plots of the NiMn*_x_*-BDC. (d) Chronopotentiometric measurement of NiMn_0.14_-BDC at 10 mA cm^−2^ for 14 h. (e) Rate capability evaluation of NiMn_0.14_-BDC. The insert shows the histogram of derived potentials. (f) Comparison of the driving potentials of electrocatalysts at a current density of 10 mA cm^−2^ for UOR. (g) 95%-iR corrected LSV curves of NiMn_0.14_-BDC under different urea concentration. (h) Urea degradation efficiency of NiMn_0.14_-BDC for different simulated urea wastewater. (i) Urea elimination rates for the first three cycles (with 0.33 M urea).

To evaluate the electrocatalytic performance of NiMn*_x_*-BDC, UOR measurements are conducted in 1.0 M KOH with 0.33 M urea by using a standard three-electrode system. The linear sweep voltammetry (LSV) plot and cyclic voltammetry (CV) curve of the control sample Mn-BDC are presented in Figure S11. As expected, it delivers extremely low electrocatalytic performance, only endowing a limiting exchange current density of 2.0 mA cm^−2^ at an applied voltage of 1.8 V. This clarifies that the Mn-BDC is almost inert for UOR. Despite the low catalytic activity of Mn-BDC in UOR, the incorporation of Mn into Ni-BDC can significantly impact on the UOR performance.  Figure 3(a) shows the LSV plots of all the as-prepared samples. After the introduction of Mn, the catalytic activity of Ni-BDC is remarkably enhanced. Among all the Mn-doped MOFs, NiMn_0.14_-BDC exhibits the highest UOR performance requiring a potential of 1.317 V to achieve a current density of 10 mA cm^−2^. The trend of Mn content affecting the UOR performance is clearly illustrated in the inset of Figure 3(a), displaying a volcano relationship between Mn/Ni ratio and UOR overpotential. This implies that the Ni and Mn sites should synergistically work for the UOR process. The electrochemically active surface area (ECSA) is determined from the electrochemical double-layer capacitance (*C*_dl_) [40]. As shown in Figure S12 and Figure 3(b), the NiMn_0.14_-BDC possesses the largest *C*_dl_ value of 2.76 mF cm^−2^, which is higher than those of the Ni-BDC (0.85 mF cm^−2^), NiMn_0.12_-BDC (0.86 mF cm^−2^), NiMn_0.13_-BDC (1.60 mF cm^−2^), NiMn_0.16_-BDC (1.94 mF cm^−2^), and NiMn_0.19_-BDC (1.78 mF cm^−2^), suggesting that more active sites are exposed in NiMn_0.14_-BDC.

To shed light on the UOR kinetics, Tafel plots are calculated as well. As expected, the NiMn_0.14_-BDC has a lower Tafel slope value of 20 mV dec^−1^ compared to that of the Ni-BDC (25 mV dec^−1^) (Figure S13), confirming that the reaction kinetics can be enhanced upon Mn incorporation. To further understand the UOR kinetics, the electrochemical impedance spectra are measured.  Figure 3(c) reveals the Nyquist plot of the as-prepared samples, whose equivalent circuit model is depicted in Figure S14, in which the *R*_ct_ represents the electrode/electrolyte interface charge transfer resistance. The detailed determined resistance values are depicted in Table S2. It can be easily discerned that *R*_ct_ of NiMn_0.14_-BDC is the smallest among all samples, confirming that a fast faradaic process is involved to yield a favorable charge-transfer kinetics. Moreover, the turnover frequency (TOF) is calculated to assess the intrinsic activity of catalysts [41]. Impressively, the TOF of NiMn_0.14_-BDC (at 1.4 V) is 0.15 s^−1^, 2.5 times higher than that of the Ni-BDC (0.06 s^−1^) (Figure S15), signifying that the incorporation of Mn can effectively enhance the intrinsic catalytic activity of Ni-BDC.

In addition to the splendid UOR activity, a chronoamperometric (*v*–*t*) measurement is conducted to evaluate the durability of NiMn_0.14_-BDC. As shown in Figure 3(d), the attenuation rate of NiMn_0.14_-BDC catalyst after 14 h measurement at a constant current density of 10 mA cm^−2^ is only 0.7%, signifying its robust long-term stability. Of note, the sawtooth oscillation in the curve could be related to the accumulation and release of N_2_ and/or CO_2_ bubbles during the UOR process. From the perspective of practical applications, the chronoamperometric measurement at different current densities is also required.  Figure 3(e) depicts the rating performance of NiMn_0.14_-BDC at current densities in the range of 10 to 90 mA·cm^−2^. Again, stable durability is observed at different current densities, and it is noticed that a high current density of 90 mA·cm^−2^ can be achieved with a relative low driving voltage of 1.50 V. Notably, our NiMn_0.14_-BDC catalyst indeed exhibits superior performance to most of the recently reported transition metal-based and the state-of-the-art precious metal-based UOR catalysts (Figure 3(f) and Table S3).

It is widely acknowledged that the OER kinetics is generally sluggish, and as such, searching for alternative anodic oxidation for low-energy consumption hydrogen generation is highly desirable. Inspired by our previous work as well as the literature reported elsewhere, UOR is a great potential candidate for anodic reaction toward hydrogen generation via water splitting [4, 30, 42]. On this occasion, the UOR and OER activity of NiMn_0.14_-BDC is evaluated. As shown in Figure S16, a potential of 1.372 V is required for the NiMn_0.14_-BDC to achieve a current density of 100 mA cm^−2^, which is 333 mV less than that of the OER (1.705 V). This manifests the possibility of UOR substitution for OER toward hydrogen generation via water splitting at low-energy consumption. Further, we appraise the UOR catalytic activity of NiMn_0.14_-BDC under different electrolyte concentrations. As shown in the LSV curves (Figure 3(g)), 1.348, 1.317, and 1.321 V potentials are required for achieving the current density of 10 mA cm^−2^ at the urea concentrations of 0.10, 0.33, and 0.50 M, respectively. Different from the general catalytic behaviors, the endowed current density is reduced when the urea concentration increases from 0.33 M to 0.50 M as the density of OH^−^ ions around the Ni active sites decreases upon rising the concentration of urea solution [43].

It should be noted that the UOR is an effective strategy for urea degradation in the industrial wastewater and domestic sewage. For comparison, 0.0033 M, 0.033 M, and 0.33 M urea solutions are selected for urea degradation. Urea degradation efficiency is measured with a modified diacetyl mono oxime-antipyrine chemical method, and the experimental details are shown in the Experimental section (Supporting Information). The effect of urea degradation by NiMn_0.14_-BDC can be qualitatively confirmed from color of the solution after catalytic treatment as shown in Figure S17. Prior to the UV absorption spectroscopy measurement, calibration is performed by establishing standard curves of urea solution with different concentrations as shown in Figure S18. By measuring the absorption features of the urea solution (Table S4), the urea degradation efficiency is determined to be 97.65%, 96.71%, and 81.87% in 0.0033 M, 0.033 M, and 0.33 M urea solution, respectively, after 3 hours of continuous working (Figure 3(h)). This demonstrates excellent urea degradation performance of the 97.65NiMn_0.14_-BDC. During the urea degradation process, fast bubbling behaviors are well observed (Movie S1). For comparison, the urea degradation efficiency of bare nickel foam is also evaluated under the same condition. The very low urea degradation effort informs that the Ni foam substrate has very limited contribution to the observed splendid urea degradation efficiency (Figure S19). A prominent urea degradation efficiency of 78.40% (with a urea concentration of 0.33 M) can be still achieved after 3 cycles of continuous working, suggesting that the as-prepared NiMn_0.14_-BDC can be repeatedly used (Figure 3(i) and Table S5). By plotting ln(*C*_0_/*C*) versus irradiation time, it can be found that the degradation of urea is associated to the quasi-first-order kinetics ln(*C*_0_/*C*) = kt, where *k* is the apparent rate constant, *C*_0_ is the initial urea concentration, and *C* is the residual urea concentration in the reaction system (Figure S20) [44]. The *k* values of NiMn_0.14_-BDC/NF in 0.0033 M, 0.033 M, and 0.33 M urea solution are calculated to be 0.028, 0.022, and 0.012 min^−1^, respectively, evidencing the fact that the high concentration of urea will reduce the reaction kinetic of urea degradation.

### 2.3. Mechanistic Study

**Figure 4:**
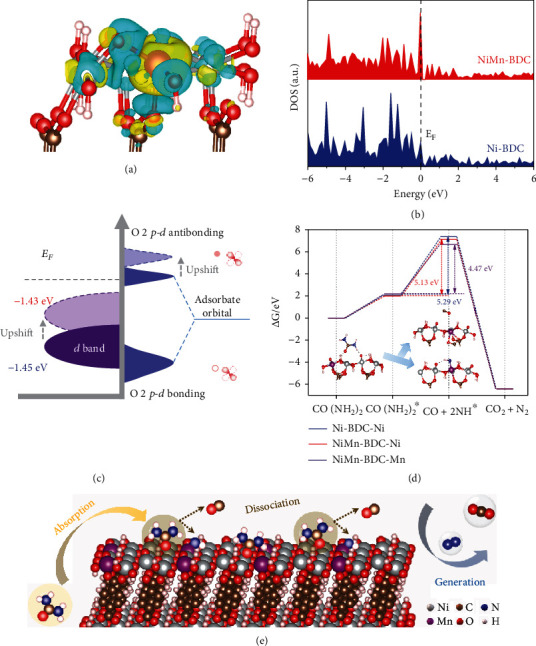
Theoretical calculations. (a) Simulated differential charge density for the NiMn-BDC. The yellow region represents charge accumulation, and the cyan region represents charge depletion. Color code: Ni (silver), Mn (purple), C (brown), O (red), and H (light pink). (b) Calculated density of states (DOS) of the Ni-BDC and NiMn-BDC. (c) Schematic diagram for the band structures of Ni-BDC and NiMn-BDC. (d) Reaction free energy profiles of the UOR on Ni-BDC and NiMn-BDC surfaces. The inset shows the corresponding structural evolution of the reaction intermediates adsorbed on the NiMn-BDC. (e) Schematic illustration for the UOR process on the surface of NiMn-BDC.

To understand the origin of the enhanced UOR performance, density functional theory (DFT) calculations are performed. The structure models of Ni-BDC and NiMn-BDC are displayed in Figure S21. To reveal the effect of Mn on the electronic structure of NiMn-BDC, the differential charge density of NiMn-BDC is simulated. As shown in Figure 4(a), substantial electrons are accumulated around the Mn atoms, while charge depletion occurs around the Ni atoms, suggesting a modulated electronic structure of metal centers after the incorporation of Mn, which is consistent with the aforementioned XPS and XANES results. The change of electronic structure is further explored by the density of state (DOS). Figure 4(b) exhibits the total DOS of Ni-BDC and NiMn-BDC. As compared with Ni-BDC, higher occupation near the Fermi level (*E*_*F*_) is observed for NiMn-BDC, indicating higher conductivity [45]. The coupling degree between the *p* orbital of the adsorbed species and the d orbital of the metal determines the strength of the adsorption energy on the surface of catalyst [19, 46]. For this reason, we further quantify the position of *d*-band center (*E*_*d*_) from the partial density of states (PDOS) of d orbitals of metals (Figure S22). The calculated *E*_*d*_ of NiMn-BDC s is -1.43 eV, closer to the *E*_*F*_ compared to that of the Ni-BDC (-1.46 eV). According to the *d*-band theory, the upshift of *E*_*d*_ implies that the unoccupied antibonding states above the Fermi level are promoted, thermodynamically strengthening the affinity of catalysts toward oxygen intermediates, which is beneficial to improve the catalytic activity of catalyst toward UOR (Figure 4(c)) [47].

Finally, to further depict the catalytic roles of Mn and Ni sites, the reaction barriers for UOR on the Ni-BDC and NiMn-BDC surfaces are calculated, including the CO(NH_2_)_2_ adsorption and the subsequent conversion of CO(NH_2_)_2_∗ to NH∗ and CO∗ intermediates (i.e., rate-determining step, RDS). The atomic structures of the reaction intermediates (CO(NH_2_)_2_∗, NH∗ and CO∗) on Ni-BDC and NiMn-BDC are displayed in Figures S23-S25. As shown in Figure 4(d) and Figure S26, the required adsorption energy of CO(NH_2_)_2_ for the Ni single sites in Ni-BDC is as large as 2.07 eV. In comparison, the counterpart energy for NiMn-BDC is reduced to 1.99 eV. This indicates that the presence of Mn sites can weaken the adsorption barrier of CO(NH_2_)_2_ at the Ni sites, which is beneficial to accelerate the kinetics of UOR. More specifically, in the case of NiMn-BDC, the adsorption energy barrier of CO(NH_2_)_2_ at Ni sites is lower than that of Mn sites (2.20 eV), suggesting that the CO(NH_2_)_2_∗ tends to be bonded with Ni sites rather than Mn sites.

Moreover, the required free energy for the RDS at the Mn sites is 4.47 eV, comparatively lower than that of the Ni sites (5.13 eV), verifying that the Mn sites serve as the highly energetic sites for the formation of key intermediates (NH∗ and CO∗). Our XPS characterization reveals that the ratio of Mn^4+^/Mn^3+^ in NiMn-BDC is reduced after UOR reactions (Figure S27). This further confirms that Mn acts as the active sites to undergo a redox reaction with urea. Overall, in the UOR process, the CO(NH_2_)_2_ is first adsorbed at the Ni sites and then cracked into NH∗ and CO∗ intermediates with the assistance of Mn sites, finally generating CO_2_ and N_2_ (Figure 4(e)).

## 3. Discussion

In summary, our rationally designed NiMn-BDC exhibits excellent UOR activity and exceptional urea degradation efficiency. Referring to the atomic-level characterizations and in-depth theoretical calculations, the enhanced catalytic performance of NiMn-BDC for UOR should be mainly attributed to the synergistic effect of bimetallic Ni-Mn centers, in which the electrons transferred from Ni to the adjacent Mn enhance the conductivity and upshift the *d*-band center of the catalyst. From the perspective of species evolution pathway, the Ni sites favor the urea absorption, and the Mn sites serve as the highly energetic sites for generating key intermediates, thereby reducing the energy requirements for UOR. This work provides new insights into the design of high-performance MOF-based catalysts for UOR at atomic precision and offers important information for understanding the UOR process at molecular level.

## 4. Materials and Methods

### 4.1. Materials and Chemicals

Nitrate salts including Ni(NO_3_)_2_·6H_2_O and Mn(NO_3_)_2_·4H_2_O and other precursors including N,N-dimethylacetamide (DMAC) and terephthalic acid (H_2_BDC) were obtained from Aladdin Company (Shanghai, China). All chemical reagents were of analytical grade and used as received without further purification.

### 4.2. Synthesis of Ni-BDC

The Ni-BDC was fabricated via a one-pot solvothermal process. In detail, 200 mg Ni(NO_3_)_2_·6H_2_O and 70 mg H_2_BDC were fully dissolved in 60 mL of DMAC to produce precursor solutions. The as-prepared solution was hydrothermally treated at 150°C in a 100 mL volume Teflon-lined stainless-steel autoclave for 3 h. Finally, the light green precipitate was obtained after being washed with DMAC and ethanol in turn and subjected to drying at 60°C in a vacuum oven.

### 4.3. Synthesis of NiMn*_x_*-BDC

The NiMn-BDC was synthesized via the same method, except for the addition of different masses of Mn(NO_3_)_2_·4H_2_O (e.g., from 40 to 120 mg with the interval of 20 mg) as a source of Mn dopant. The as-obtained samples were named as NiMn*_x_*-BDC (where *x* represents the molar ratio of Mn:Ni).

### 4.4. Synthesis of Mn-BDC

For purpose of comparison, a control sample of bare Mn-BDC was prepared under identical conditions to Ni-BDC, except that 200 mg of Mn(NO_3_)_2_·4H_2_O was added instead of the Ni(NO_3_)_2_·6H_2_O precursor.

### 4.5. Electrochemical Measurements

To prepare a working electrode, a specific amount of the catalyst (5.0 mg), conductive acetylene black (1.0 mg), and Nafion solution (30 *μ*L) was mixed in 1 mL solution of water/ethanol (1 : 1) under ultrasonic treatment, and then, 10 *μ*L of the catalyst ink was dropped onto the glassy carbon (GC) electrode which served as a working electrode during electrochemical measurements. An electrochemical potential was set up by employing Ag/AgCl electrode as a reference and carbon rod as a counter electrode. The electrocatalytic experiments were performed in a three-electrode configuration system connected to a CHI 760E electrochemical workstation (Chenhua Instruments, Shanghai). Further, the electrochemical urea oxidation reactions were performed in a 1.0 M KOH containing 0.33 M urea as the aqueous electrolyte, and the rotation speed was maintained at 1600 rpm. The linear sweep voltammetry (LSV) experiments were recorded at potential values of -0.023 to 0.777 V *vs.* the saturated Ag/AgCl electrode at a scan rate of 5 mV/s, with 95% *iR* correction to compensate for the electrolyte resistance. Chronopotentiometry was performed under a constant current density of 10 mA/cm^2^. Tafel slope was determined by the following equation: *η* = blog(*j*) + *a*, where *η* is the overpotential, *b* the Tafel slope, and *j* the current density. Electrochemical impedance spectroscopy (EIS) measurements were performed at a frequency range of 0.1 Hz to100 kHz. For measuring the electrochemically active surface areas (ECSA), the potential was set in the range of 0.06 V to 0.16 V *vs.* the Ag/AgCl electrode, and the scan rate was 10 mV/s to 50 mV/s with interval of 10 mV/s. The turnover frequency (TOF) of catalysts was calculated according to the equation: TOF = *J*∗*A*/6∗*F*∗*n* TOF = *J*∗*A*/6∗*F*∗*n* TOF = *J*∗*A*/6∗*F*∗*n*, where *J* is the current density at 1.4 V, *A* the electrode surface area, *F* is the faradaic constant, and *n* is the mole number of total Ni sites on the electrode. The *d*-band center values of Ni-BDC and NiMn-BDC are conducted as ∫*N*(*ε*)*εd*_*ε*_/∫*N*(*ε*)*d*_*ε*_ in the range of -8.0 ~ 4.0 eV.

For the urea degradation measurements, the catalyst (30 mg), conductive acetylene black (1.0 mg) and Nafion solution (30 *μ*L) were mixed in 1 mL of water/ethanol solution (1 : 1) under ultrasonic treatment. The catalyst ink was dropped onto the Ni foam (NF) (2 cm × 4 cm), which was employed as the electrode and labeled as NiMn_0.14_-BDC/NF. The NiMn_0.14_-BDC/NF was used as the anode and cathode under the condition of the constant pressure of 5 V. Take the remaining urea solution every 20 min, the degradation rate of urea was measured by a modified diacetyl mono oxime-antipyrine chemical method with a UV spectrophotometer. The urea concentration was determined by a modified diacetyl monoxime method. 2.0 g of 4-aminoantipyrine and 0.25 g of FeCl_3_·6H_2_O were dissolved in deionized water and diluted to 1000 mL; then, 100 mL of concentrated sulfuric acid and 200 mL of concentrated phosphoric acid were added in slowly, which is termed as the solution A. 1.0 g of diacetyl monooxime was dissolved in 50 mL of acetic acid solution (*V*_acetic acid_ : *V*_deionized_ water = 1 : 9), and then, 50 mL of isopropanol was added to the above solution slowly (termed as the solution B). Noteworthy, 1 mL of the sample solution was generally mixed with 1.0 mL sulfuric acid (1 M) and diluted to 10 mL. In our case, 4.0 mL of solution A and 2 mL of solution B were added into the sample-sulfuric acid solution, forming aubergine solution. Next, the mixed solution was heated to 100°C until the solution turned yellow. When the solution was cooled down to room temperature, the UV-Vis absorption spectrum was recorded at a wavelength of 460 nm. The degradation efficiency (DE) was calculated according to the equation: DE = 1 − *C*/*C*_0_, where *C* is the residual urea concentration and *C*_0_ is the initial urea concentration.

### 4.6. Computational Methods

To explore the geometries and reaction paths of the UOR catalyzed by the NiMn-BDC, spin-polarized density-function-theory (DFT) calculations were achieved via the Vienna ab initio simulation package (VASP) program package [48, 49] within the projector augmented wave (PAW) [50]. The exchange-correlation interactions were designated with the generalized-gradient-approximation (GGA) [51] in the form of Perdew, Burke, and Ernzernhof (PBE) functional [52]. The Ni-BDC was constructed according to a previous study [53]. The NiMn-BDC structure was derived by replacing one Ni atom with Mn atom. A 500 eV cutoff kinetic energy was set for the plane-wave basis, and vacuum layer distance was set to be greater than 15 Å, which was adequate to avoid the interlayer interactions. The DFT-D3 scheme of Grimme for the vdW correction [54] was applied on MOF surface. The electronic SCF tolerance was fixed as 10^−5^ eV. Fully relaxed geometries and lattice constant were obtained by optimizing all the atomic positions until the Hellmann–Feynman forces were less than 0.02 eV/Å. The *k*-point samplings with a gamma-centered Monkhorst-Pack scheme [55] were 3 × 5 × 1 for structural optimizations. The reaction free energy diagram with the formation of NH∗ was obtained as a simplified three-state diagram comprising an initial state (adsorbed CO(NH_2_)_2_∗), an intermediate state (NH∗ and CO adsorbed in gas phase), and a final state (product CO_2_ and N_2_) [56].

### 4.7. Characterization

The phases of as-fabricated MOFs were analyzed via X-ray powder diffraction (XRD) using an Empyrean X'pert Pro X-ray diffractometer (Philips, Cu K*α*, *λ* = 1.5406 Å). The morphological structures of the as-fabricated MOFs were analyzed via scanning electron microscopy (SEM; ZEISS G300) together with corresponding elemental mapping and transmission electron microscopy (TEM; Tecnai, G2-F30). The crystallinity of the materials was identified via high-resolution transition-electron microscopy (HR-TEM) and selected-area electron diffraction (SAED). A Kratos-AXIS Ultra DLD-600W X-ray photoelectron spectrometer (XPS; Al K*α* (1486.6 eV) X-ray source) was employed to examine the surface chemical states of the catalysts. The content of the elements in the catalysts was identified via the inductively coupled plasma-optical emission spectrometer (ICP-OES, Agilent ICP-OES 720). EXAFS and XANES of Ni and/or Mn *K*-edge were performed at BL01C1 beamline of National Synchrotron Radiation Reaction Center (NSRRC) to explore the electronic structures of catalysts. The BET surface area and BJH pore volumes were estimated by the analyses of N2 adsorption-desorption isotherms (TriStar 20). The urea degradation rate was detected by ultraviolet-visible spectrophotometer (721-100).

## Data Availability

All data needed to evaluate the conclusions in the paper are present in the paper and/or the Supplementary Materials. Additional data related to this paper may be requested from the authors.

## Supplementary Materials

Supplementary Materials Figure S1. (a) Weight ratios of Ni and Mn for various NiMn-BDC electrodes. (b) Molar ratios of Mn:Ni for various NiMn-BDC electrodes. Figure S2. Photographs of (a) Ni-BDC, (b) NiMn0.12-BDC, (c) NiMn0.13-BDC, (d) NiMn0.14-BDC, (e) NiMn0.16-BDC, and (f) NiMn0.19-BDC. Figure S3. SEM images of (a) Ni-BDC, (b) NiMn0.12-BDC, (c) NiMn0.13-BDC, (d) NiMn0.16-BDC, (e) NiMn0.19-BDC, and (f) Mn-BDC. Figure S4. HRTEM image of Ni-BDC. Figure S5. (a) XRD patterns of the as-prepared Ni-BDC and the simulated Ni-BDC MOF. (b) FT-IR spectra of Ni-BDC and NiMn0.14-BDC. Figure S6. X-band EPR spectra of Ni-BDC and NiMn0.14-BDC. Figure S7. (a) BET surface area and (b) pore size distribution of Ni-BDC and NiMn0.14-BDC. Figure S8. (a) XPS survey spectrum of NiMn0.14-BDC. (b) High-resolution O 1s XPS spectra of Ni-BDC and NiMn0.14-BDC. Figure S9. EXAFS spectra of Ni-BDC, NiMn0.14-BDC, and reference materials at the Mn K-edge. Figure S10. The Ni K-edge EXAFS fitting results of Ni-O bonds for Ni-BDC at (a) R space and (b) K space. The Ni K-edge EXAFS fitting results of Ni-O bonds for NiMn0.14-BDC at (c) R space and (d) K space. The Mn K-edge EXAFS fitting results of Mn-O bonds for NiMn0.14-BDC at (e) R space and (f) K space. Figure S11. (a) 95%-iR corrected LSV curves and (b) CV curves of Mn-BDC in 1 M KOH+0.33 M urea. Figure S12. CV curves with different scan rates of (a) Ni-BDC, (b) NiMn0.12-BDC, (c) NiMn0.13-BDC, (d) NiMn0.14-BDC, (e) NiMn0.16-BDC, and (f) NiMn0.19-BDC. Figure S13. Tafel plots of the obtained samples in 1 M KOH electrolyte with 0.33 M urea. Figure S14. Equivalent circuit model toward Nyquist plots of catalysts. Figure S15. TOF plots at a cell voltage of 1.4 V for Ni-BDC and NiMn0.14-BDC. Figure S16. 95%-iR corrected LSV curves of NiMn0.14-MOF in 1 M KOH electrolyte with and without 0.33 M urea. Figure S17. The related color changes of urea solution with an initial concentration of (a) 0.0033 M, (b) 0.033 M and (c) 0.33 M degraded at different times. Figure S18. Standard curves of urea solution with different concentrations determined by modified diacetyl mono oxime-antipyrine chemical method. Figure 19. The urea degradation efficiency of the nickel foam in urea solution with concentrations of 0.0033 M, 0.033 M and 0.33 M. Figure S20. The linear plot of ln(C0/C) versus irradiation time of NiMn0.14-BDC under different urea concentration. Figure 21. (a) Top view and (b) side view of the DFT-optimized structures of Ni-BDC. (c) Top view and (d) side view of the DFT-optimized structures of NiMn-BDC. Figure 22. The calculated partial density of states (PDOS) of d orbitals for (a) Ni-BDC and (b) NiMn-BDC. Figure 23. The corresponding structural evolution of UOR reaction intermediates adsorbed at the Ni in Ni-BDC. Figure 24. The corresponding structural evolution of UOR reaction intermediates adsorbed at the Ni in NiMn-BDC. Figure 25. The corresponding structural evolution of UOR reaction intermediates adsorbed at the Mn in NiMn-BDC. Figure S26. Adsorption energy of urea (left) and adsorption energy change from CO(NH2)2∗ to NH∗ and CO∗ intermediates (dissociation energy of CO(NH2)2∗) (right) at the Ni in NiMn-BDC, the Ni in Ni-BDC ,and the Mn in NiMn-BDC. Figure S27. XPS analysis of NiMn0.14-BDC after UOR stability test. Movie S1. Electrochemical-driven urea degradation. Table S1. Structural parameters of Ni-BDC and NiMn0.14-BDC. Fourier-transforms (FT) are performed in the K-range between 0 Å-1 and 12 Å-1. Table S2. The fitted EIS resistance of NiMn*_x_*-BDC. Table S3. UOR performance of NiMn0.14-BDC and the recently reported Ni-based and Mn-based electrocatalysts. Table S4. The absorbance value of urea solutions. Table S5. The absorbance value of NiMn0.14-BDC after different cycles.
