# Supplementary material for: Metal-Organic Frameworks Offering Tunable Binary Active Sites toward Highly Efficient Urea Oxidation Electrolysis
Source: Research (Wash D C). 2022 Jun 27;2022:9837109. doi: 10.34133/2022/9837109 (PMC9275073; doi:10.34133/2022/9837109)
Supplement: Supplementary Materials — Figure S1. (a) Weight ratios of Ni and Mn for various NiMn-BDC electrodes. (b) Molar ratios of Mn:Ni for various NiMn-BDC electrodes. Figure S2. Photographs of (a) Ni-BDC, (b) NiMn0.12-BDC, (c) NiMn0.13-BDC, (d) NiMn0.14-BDC, (e) NiMn0.16-BDC, and (f) NiMn0.19-BDC. Figure S3. SEM images of (a) Ni-BDC, (b) NiMn0.12-BDC, (c) NiMn0.13-BDC, (d) NiMn0.16-BDC, (e) NiMn0.19-BDC, and (f) Mn-BDC. Figure S4. HRTEM image of Ni-BDC. Figure S5. (a) XRD patterns of the as-prepared Ni-BDC and the simulated Ni-BDC MOF. (b) FT-IR spectra of Ni-BDC and NiMn0.14-BDC. Figure S6. X-band EPR spectra of Ni-BDC and NiMn0.14-BDC. Figure S7. (a) BET surface area and (b) pore size distribution of Ni-BDC and NiMn0.14-BDC. Figure S8. (a) XPS survey spectrum of NiMn0.14-BDC. (b) High-resolution O 1s XPS spectra of Ni-BDC and NiMn0.14-BDC. Figure S9. EXAFS spectra of Ni-BDC, NiMn0.14-BDC, and reference materials at the Mn K-edge. Figure S10. The Ni K-edge EXAFS fitting results of Ni-O bonds for Ni-BDC at (a) R space and (b) K space. The Ni K-edge EXAFS fitting results of Ni-O bonds for NiMn0.14-BDC at (c) R space and (d) K space. The Mn K-edge EXAFS fitting results of Mn-O bonds for NiMn0.14-BDC at (e) R space and (f) K space. Figure S11. (a) 95%-iR corrected LSV curves and (b) CV curves of Mn-BDC in 1 M KOH+0.33 M urea. Figure S12. CV curves with different scan rates of (a) Ni-BDC, (b) NiMn0.12-BDC, (c) NiMn0.13-BDC, (d) NiMn0.14-BDC, (e) NiMn0.16-BDC, and (f) NiMn0.19-BDC. Figure S13. Tafel plots of the obtained samples in 1 M KOH electrolyte with 0.33 M urea. Figure S14. Equivalent circuit model toward Nyquist plots of catalysts. Figure S15. TOF plots at a cell voltage of 1.4 V for Ni-BDC and NiMn0.14-BDC. Figure S16. 95%-iR corrected LSV curves of NiMn0.14-MOF in 1 M KOH electrolyte with and without 0.33 M urea. Figure S17. The related color changes of urea solution with an initial concentration of (a) 0.0033 M, (b) 0.033 M and (c) 0.33 M degraded at different times. Figure S18. Standard curv [file 9837109.f1.zip › NiMn-BDC-Research-SI-revised-clean.pdf]

Research
A SCIENCE PARTNER JOURNAL
SPJ
SCIENCE PARTNER JOURNALS

Supporting Information

Title

Metal-Organic Frameworks Offering Tunable Binary Active Sites toward Highly Efficient Urea Oxidation Electrolysis

Authors

Xuefei Xu[1]†, Qingming Deng[2]†, Hsiao-Chien Chen[3]†, Muhammad Humayun[1], Delong Duan[4], Xia Zhang[1], Huachuan Sun[1], Xiang Ao[1], Xinying Xue[5], Anton Nikiforov[6], Kaifu Huo[1], Chundong Wang[1*], Yujie Xiong[4*]

Affiliations

[1]School of Optical and Electronic Information, Wuhan National Laboratory for Optoelectronics, Optics Valley Laboratory, Huazhong University of Science and Technology, Wuhan 430074, P. R. China
[2]Physics Department and Jiangsu Key Laboratory for Chemistry of Low-Dimensional Materials, Huaiyin Normal University, Huaian 223300, P. R. China
[3]Center for Reliability Science and Technologies, Chang Gung University, Taoyuan 33302, Taiwan, China
[4]School of Chemistry and Materials Science, University of Science and Technology of China, Hefei 230026, Anhui, P. R. China
[5]Department of Physics, College of Science, Shihezi University, Xinjiang 832003, P. R. China
[6]Department of Applied Physics, Ghent University, Gent 9000, Belgium

[†] These authors contributed equally to this work

Correspondence should be addressed to Chundong Wang; apcdwang@hust.edu.cn and Yujie Xiong; yjxiong@ustc.edu.cn

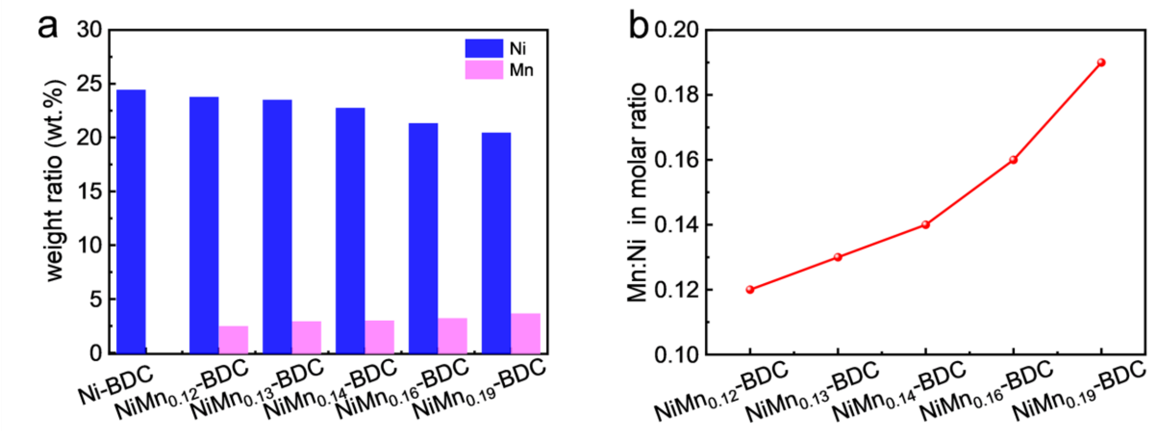

Figure S1. (a) Weight ratios of Ni and Mn for various NiMn-BDC electrodes. (b) Molar ratios of Mn:Ni for various NiMn-BDC electrodes.

The ICP-OES results in Figure S1 show that the Ni content in NiMn-BDC decreas with the increase of $Mn(NO_3)_2$ feeding. When the feeding of $Mn(NO_3)_2$ is 40, 60, 80, 100 and 120 mg, the molar ratio of Mn to Ni in NiMn-BDC is 0.12, 0.13, 0.14, 0.16 and 0.19 respectively (Figure S2).

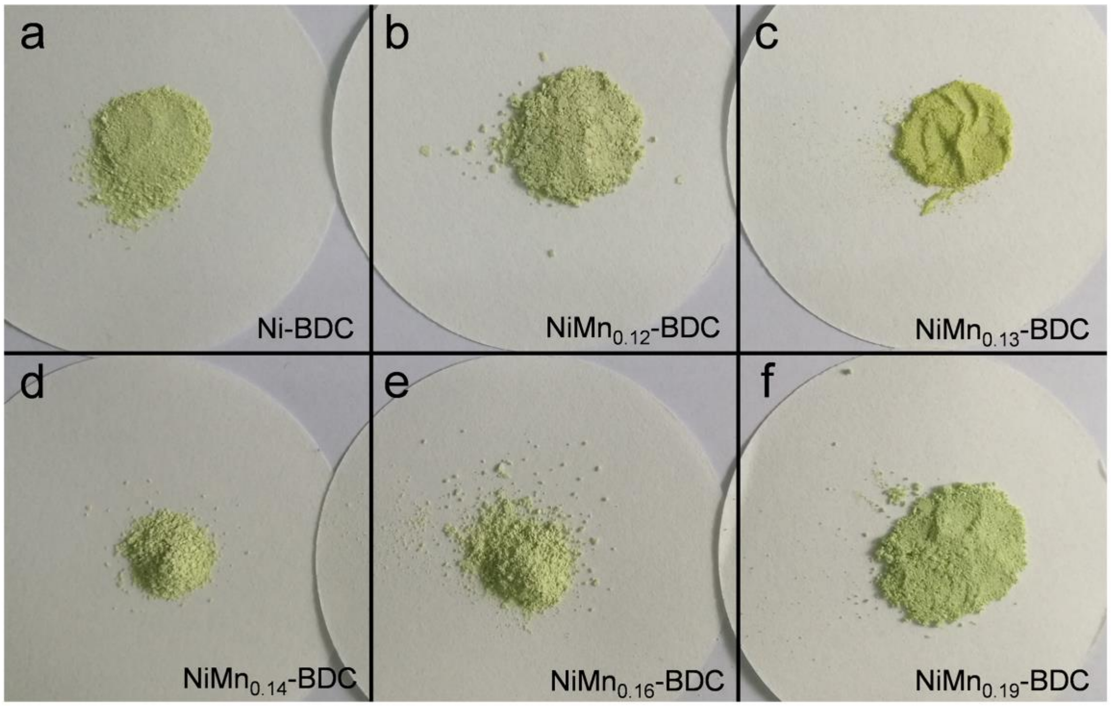

Figure S2. Photographs of (a) Ni-BDC, (b) $NiMn_{0.12}$-BDC, (c) $NiMn_{0.13}$-BDC, (d) $NiMn_{0.14}$-BDC, (e) $NiMn_{0.16}$-BDC and (f) $NiMn_{0.19}$-BDC.

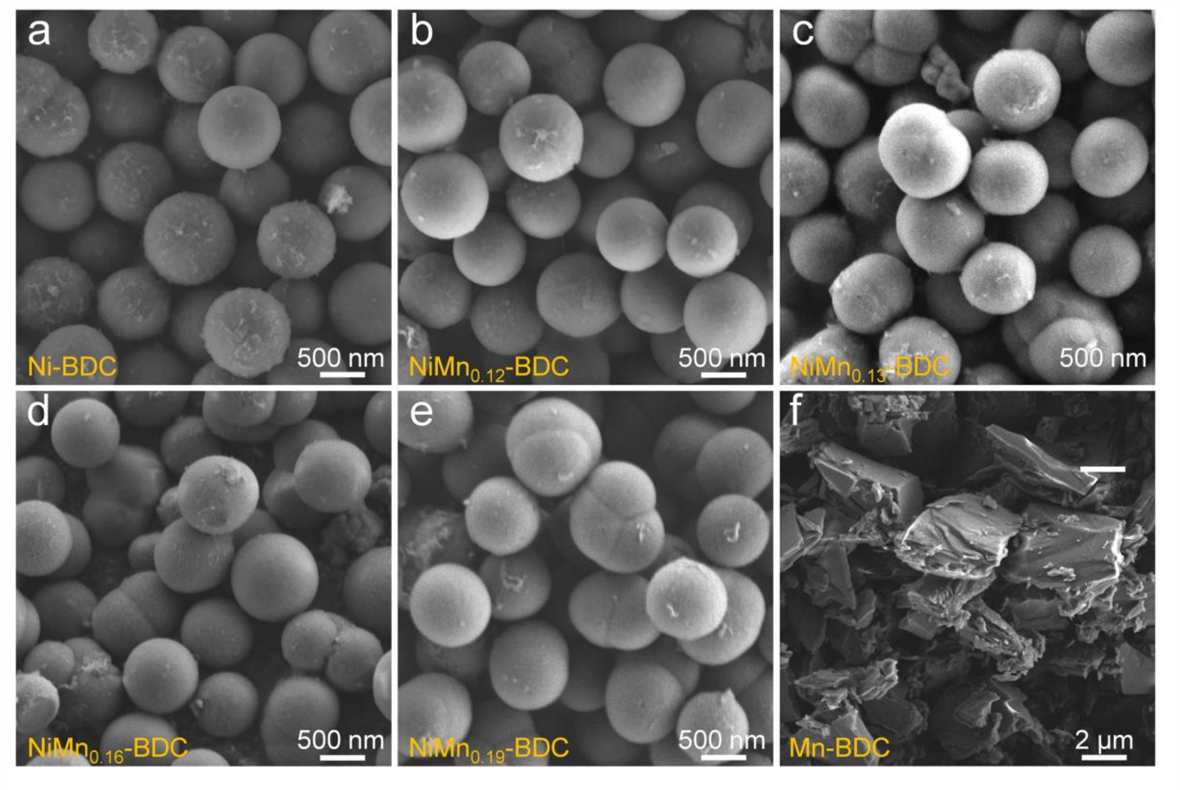

Figure S3. SEM images of (a) Ni-BDC, (b) $NiMn_{0.12}$-BDC, (c) $NiMn_{0.13}$-BDC, (d) $NiMn_{0.16}$-BDC, (e) $NiMn_{0.19}$-BDC and (f) Mn-BDC.

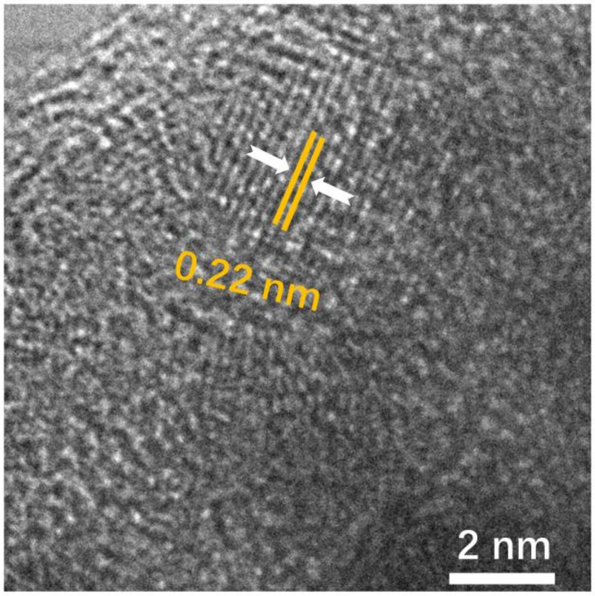

Figure S4. HRTEM image of Ni-BDC.

The fringe spacing of Ni-BDC is 0.22 nm, well indexed to the (410) plane of Ni-BDC MOF ($Ni_2(OH)_2(C_8H_4O_4)$).

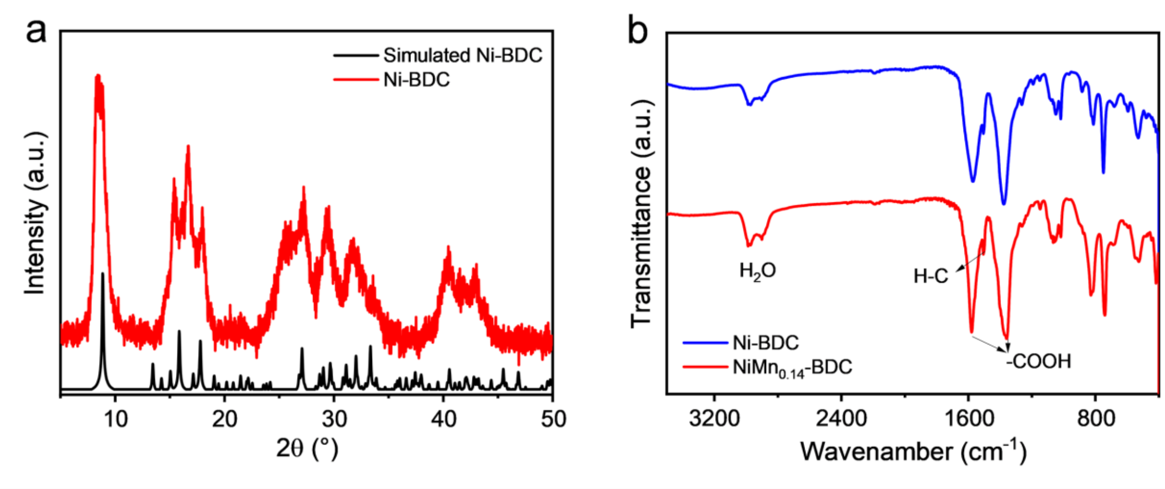

Figure S5. (a) XRD patterns of the as-prepared Ni-BDC and the simulated Ni-BDC MOF. (b) FT-IR spectra of Ni-BDC and $NiMn_{0.14}$-BDC.

The information for ligands in Ni-BDC and $NiMn_{0.14}$-BDC is obtained by the Fourier transform infrared (FT-IR) spectra. As shown in Figure S5b, the bands between 2900 cm^{-1} and 3000 cm^{-1} are associated to the stretching vibrations of water molecule, the absorption peak at 1500 cm^{-1} corresponds to the vibration of the para-aromatic C-H group, and the peaks at 1575 cm^{-1} and 1384 cm^{-1} are assigned to the asymmetric and symmetric vibrations of the coordinated carboxyl (-COO-) group, respectively [1].

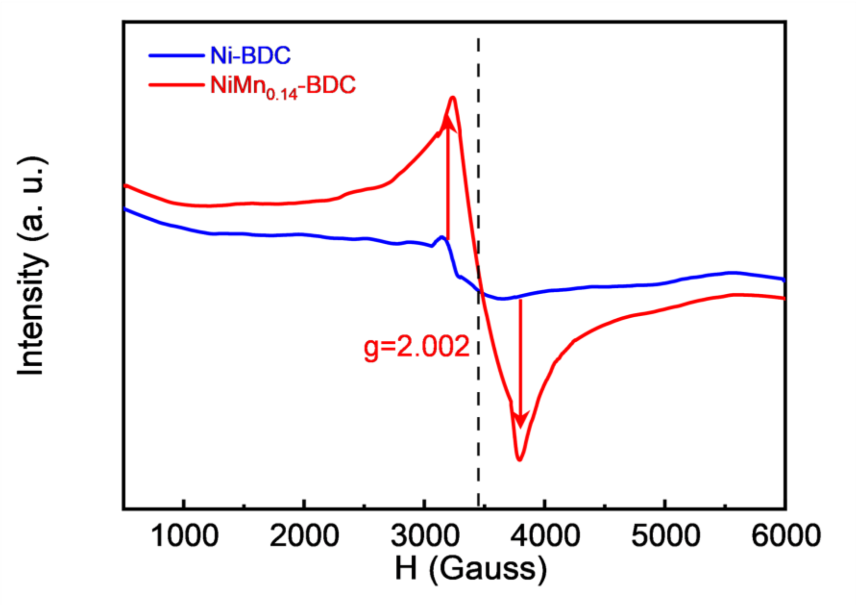

Figure S6. X-band EPR spectra of Ni-BDC and $NiMn_{0.14}$-BDC.

The signals at g = 2.002 in EPR spectrum could be identified as the electrons trapped on oxygen vacancies. The EPR spectrum of $NiMn_{0.14}$-BDC displayes a more pounced symmetric peak at g=2.002 than Ni-BDC, faithfully validating that more oxygen vacancies are created in $NiMn_{0.14}$-BDC due to the Mn incorporation.

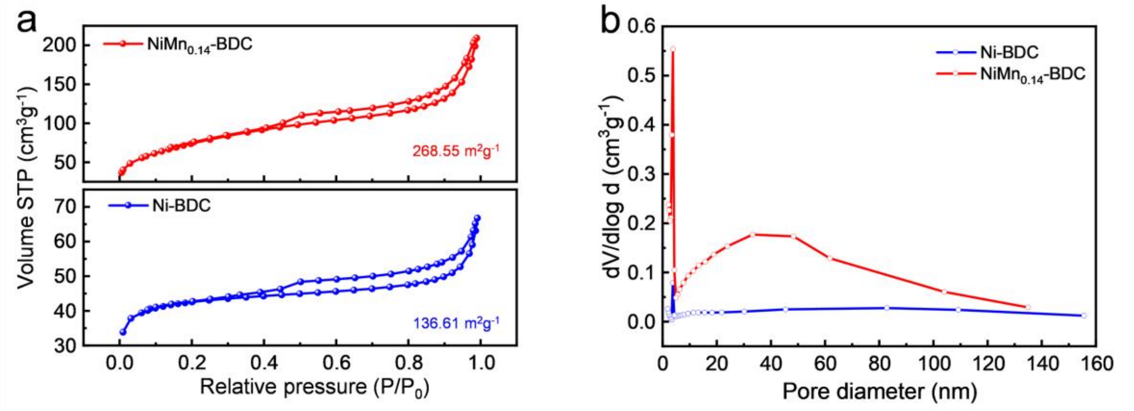

Figure S7. (a) BET surface area and (b) pore size distribution of Ni-BDC and $NiMn_{0.14}$-BDC.

N_2 adsorption and desorption isotherms (Figure S6(a)) reveal that the Brunauer–Emmett–Teller (BET) surface areas of Ni-BDC and $NiMn_{0.14}$-BDC are 136.61 and 265.55 m^2 g^{-1}. Pore size (Figure S6(b)) of Ni-BDC are mainly distributed around 4 nm, manifesting its mesoporous structure. After Mn intrducing, the pore with size of around 40 nm appeared in $NiMn_{0.14}$-BDC, indicating that the incorporation of Mn nodes can improve the amount of mesoporous in MOF structure, which allowing the metal sites exposed to reaction species, thereby enhancing UOR performance.

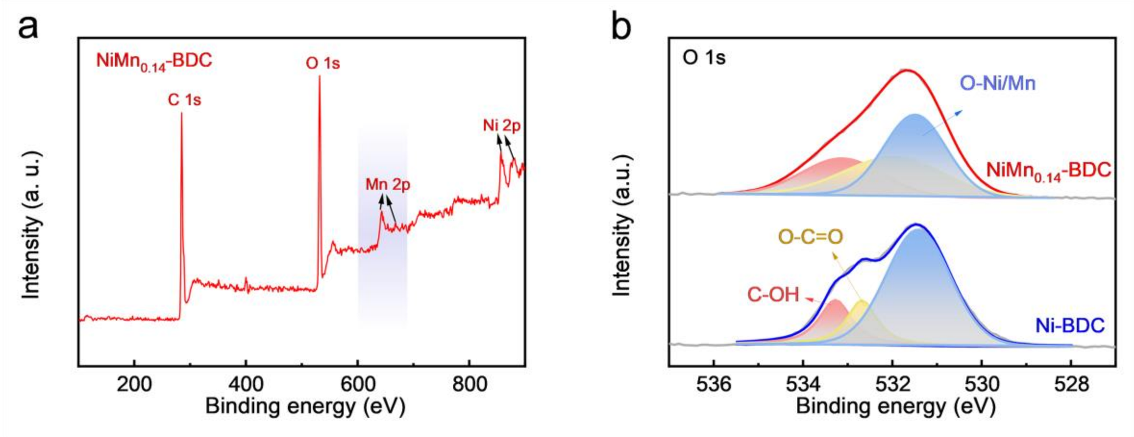

Figure S8. (a) XPS survey spectrum of $NiMn_{0.14}$-BDC. (b) High-resolution O 1s XPS spectra of Ni-BDC and $NiMn_{0.14}$-BDC.

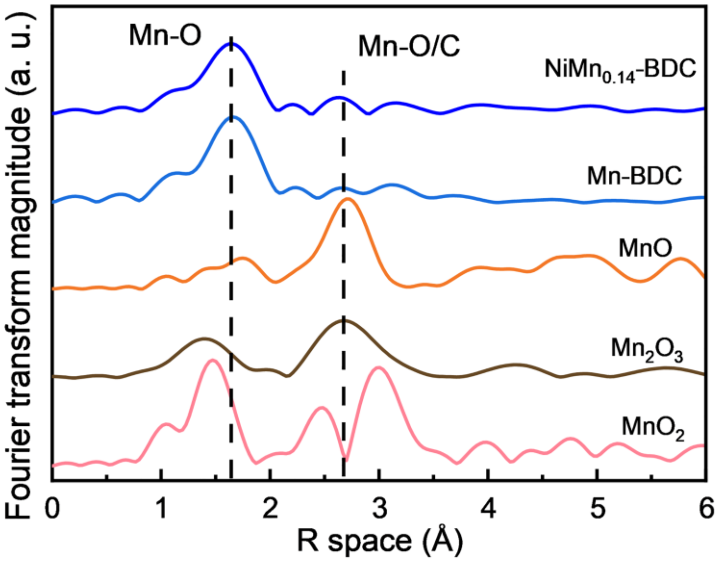

Figure S9. EXAFS spectra of Ni-BDC, $NiMn_{0.14}$-BDC and reference materials at the Mn *K*-edge.

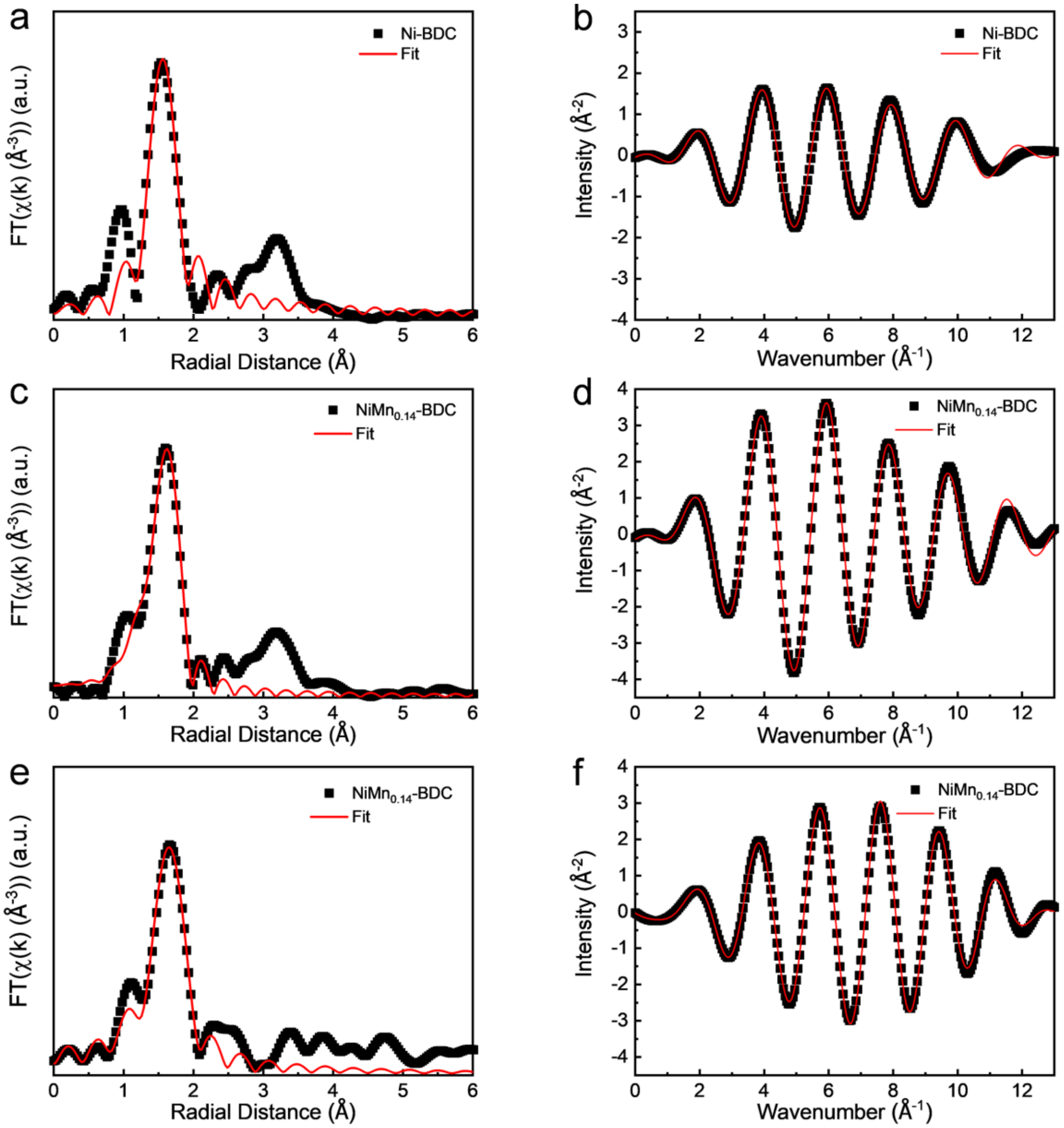

Figure S10. The Ni *K*-edge EXAFS fitting results of Ni-O bonds for Ni-BDC at (a) R space and (b) K space. The Ni *K*-edge EXAFS fitting results of Ni-O bonds for $NiMn_{0.14}$-BDC at (c) R space and (d) K space. The Mn *K*-edge EXAFS fitting results of Mn-O bonds for $NiMn_{0.14}$-BDC at (e) R space and (f) K space.

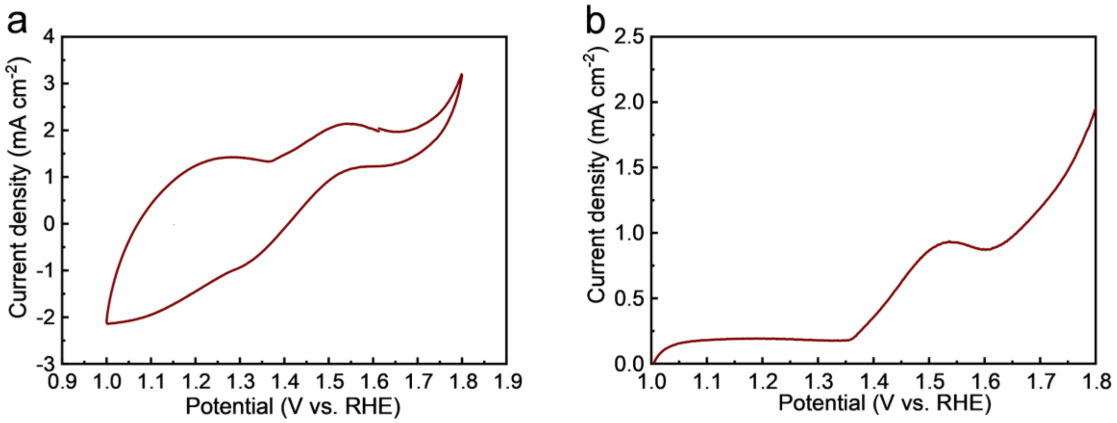

Figure S11. (a) 95%-iR corrected LSV curves and (b) CV curves of Mn-BDC in 1 M KOH + 0.33 M urea.

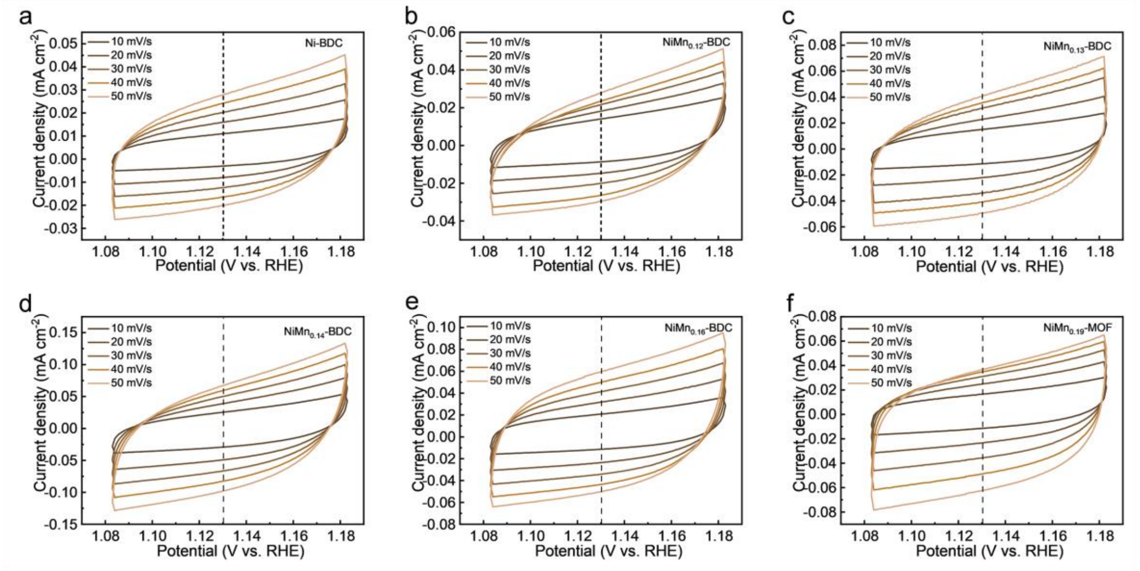

Figure S12. CVcurves with different scan rates of (a) Ni-BDC, (b) $NiMn_{0.12}$-BDC, (c) $NiMn_{0.13}$-BDC, (d) $NiMn_{0.14}$-BDC, (e) $NiMn_{0.16}$-BDC and (f) $NiMn_{0.19}$-BDC.

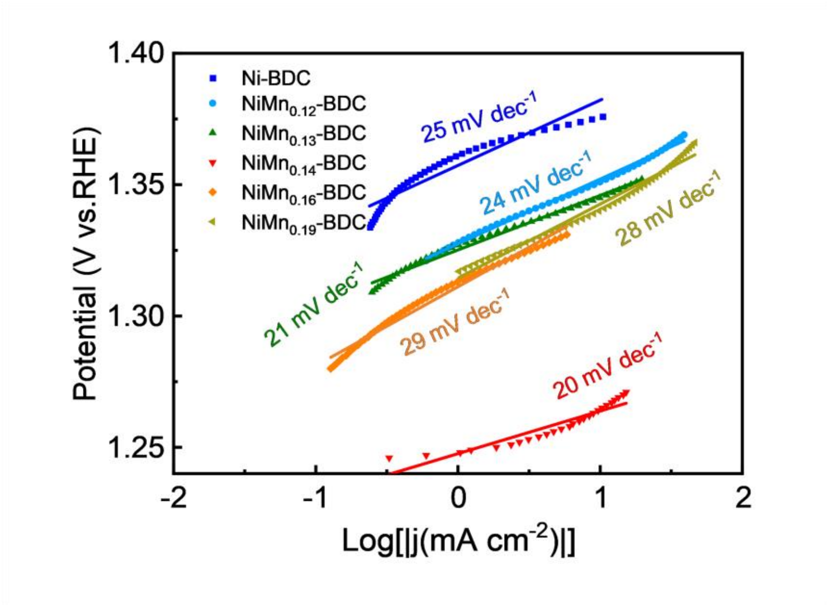

Figure S13. Tafel plots of the obtained samples in 1 M KOH electrolyte with 0.33 M urea.

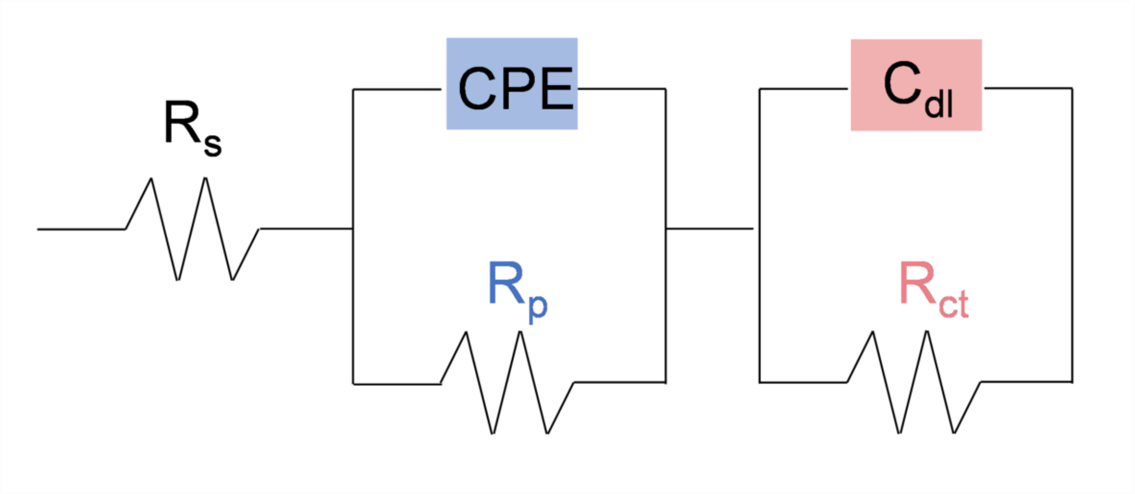

Figure S14. Equivalent circuit model toward Nyquist plots of catalysts. R_s, R_p, R_{ct}, CPE and C_{dl} denote the system resistance, electrode porosity resistance, charge transfer resistance, constant phase element and double-layer capacitance, respectively.

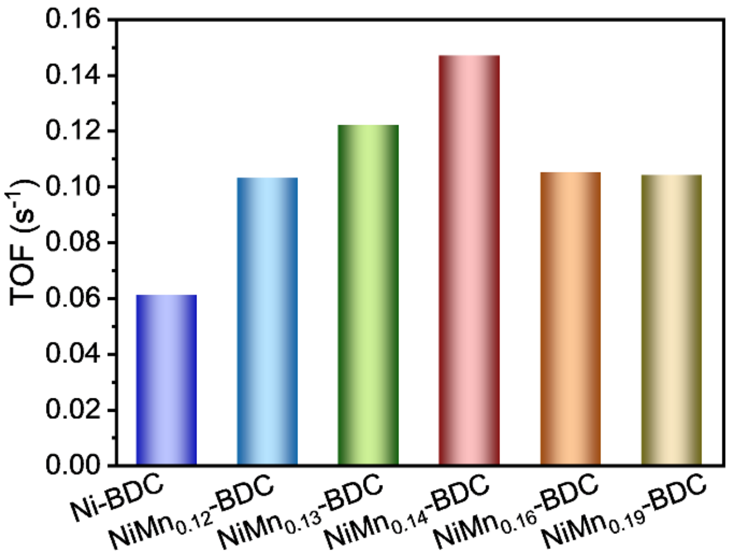

Figure S15. TOF plots at a cell voltage of 1.4 V for Ni-BDC and $NiMn_{0.14}$-BDC.

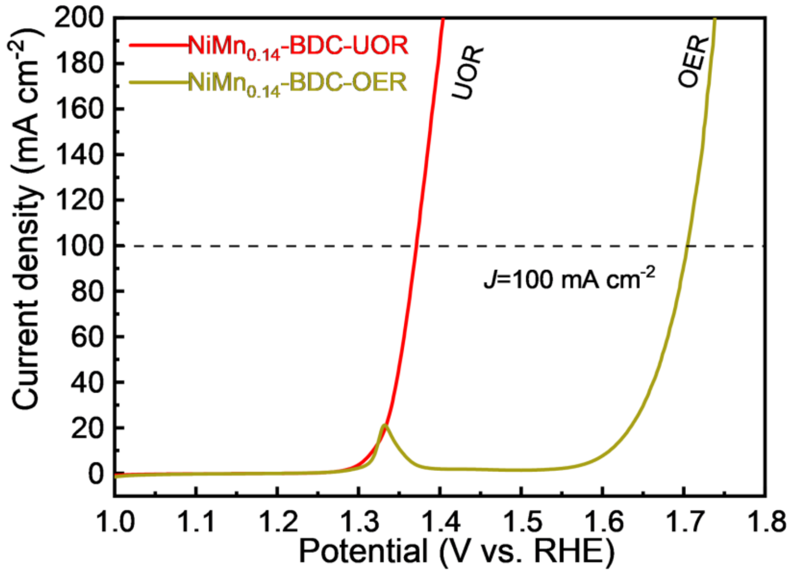

Figure S16. 95%-iR corrected LSV curves of $NiMn_{0.14}$-MOF in 1 M KOH electrolyte with and without 0.33 M urea.

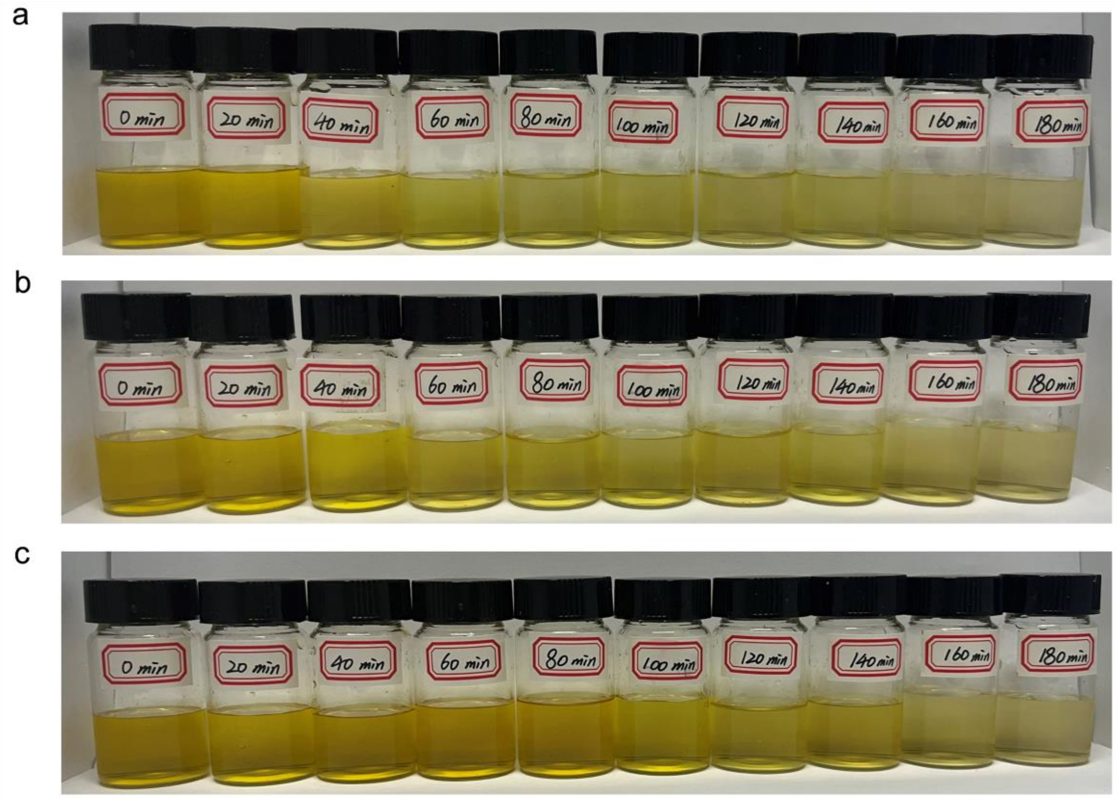

Figure S17. The related color changes of urea solution with an initial concentration of (a) 0.0033 M, (b) 0.033 M and (c) 0.33 M degraded at different times.

The coloring solutions are prepared from residual urea solution that has been degraded after different periods (Color development principle: under strong acid and heating conditions, urea reacts with diacetyl mono oxime and antipyrine to appear yellow).

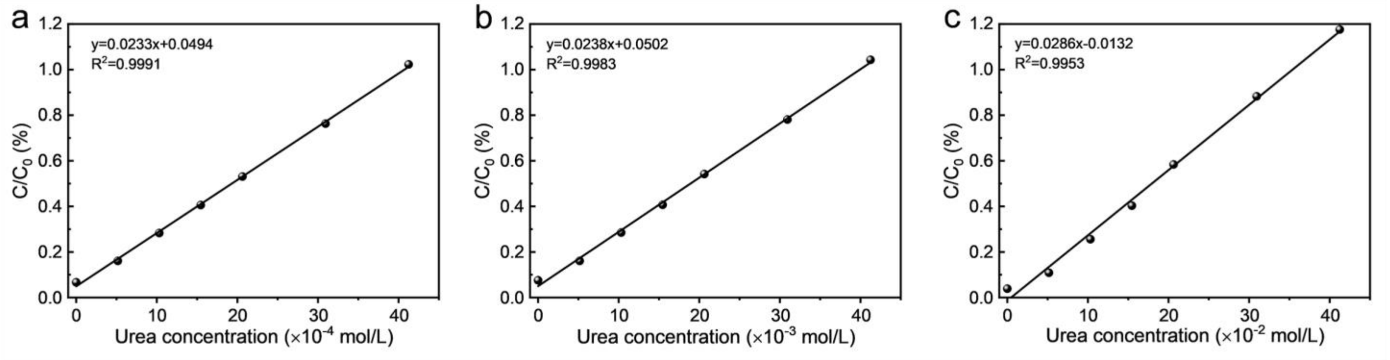

Figure S18. Standard curves of urea solution with different concentrations determined by modified diacetyl mono oxime-antipyrine chemical method.

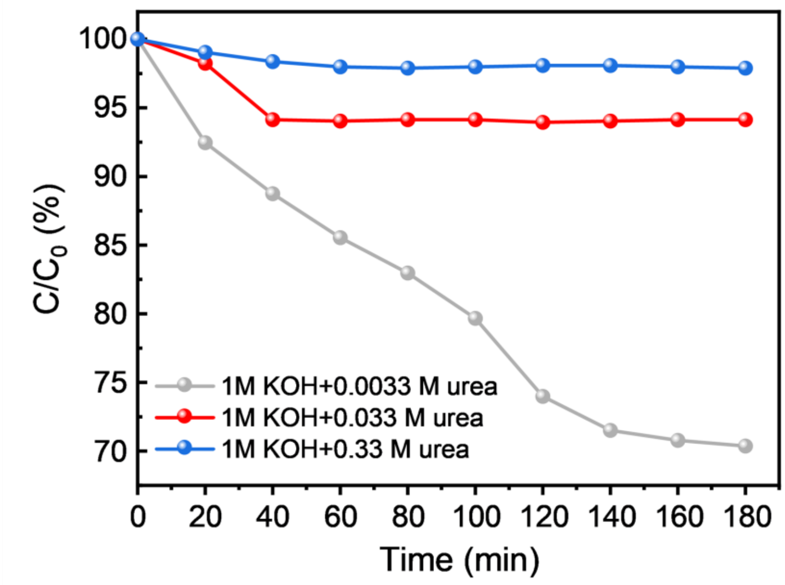

Figure S19. The urea degradation efficiency of the nickel foam in urea solution with concentrations of 0.0033 M, 0.033 M and 0.33 M.

The pure nickel foam displayed limited urea degradation efficiency of 29.63%, 5.87 and 2.12% in 0.0033 M, 0.033 M and 0.33 M urea solution, respectively, after 3 hours of continuous working. This result proving that the excellent urea degradation efficiency of $NiMn_{0.14}$-BDC/NF electrode is mainly comes from $NiMn_{0.14}$-BDC rather than the nickel foam support.

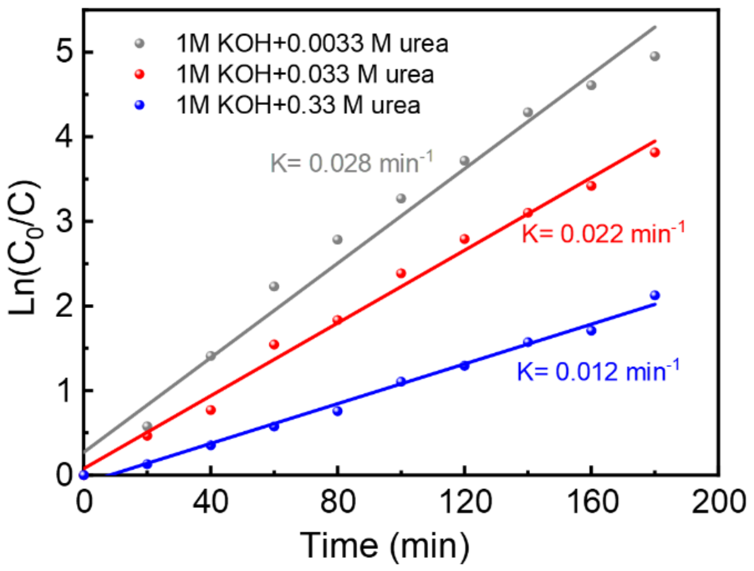

Figure S20. The linear plot of ln(C_0/C) versus irradiation time for $NiMn_{0.14}$-BDC under different urea concentrations.

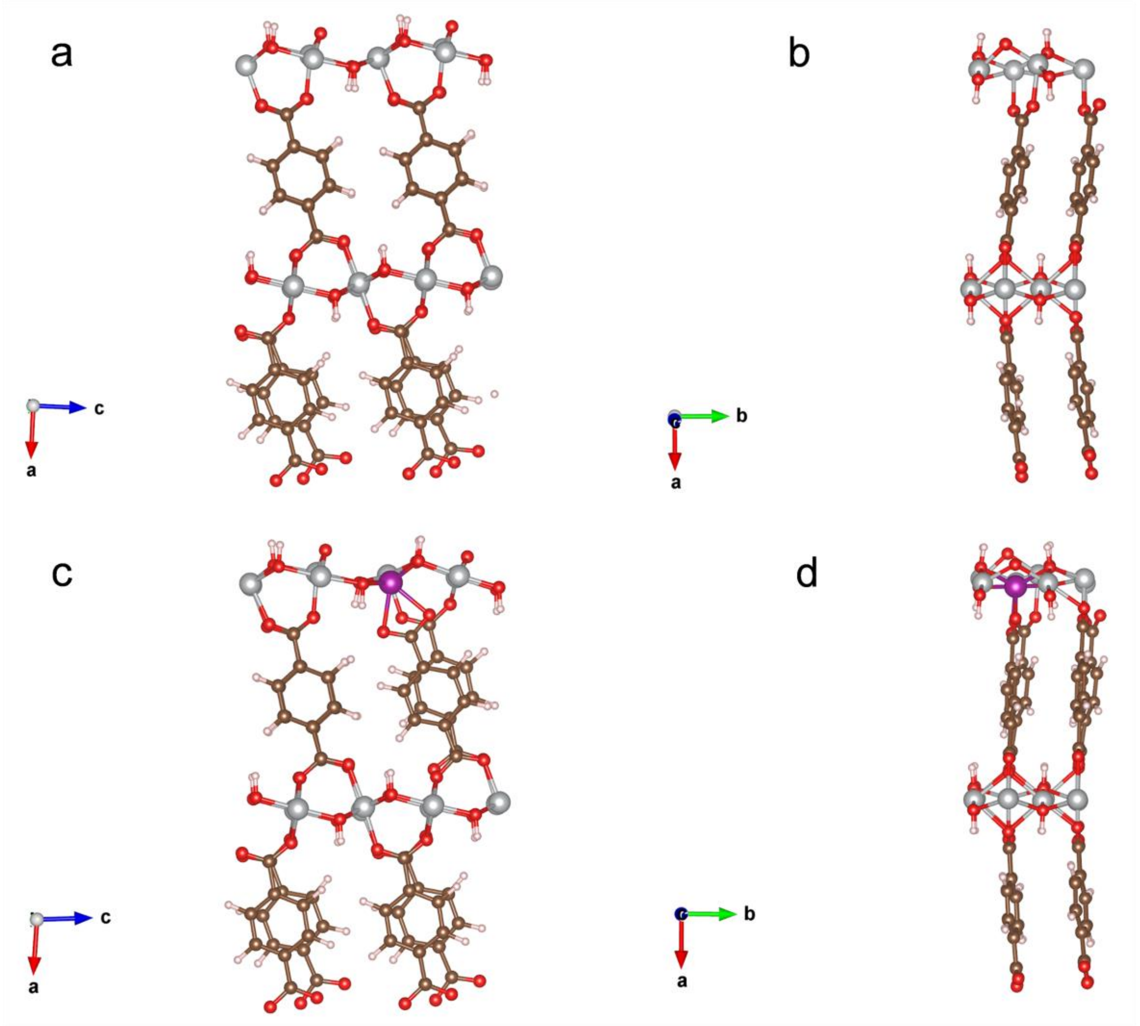

Figure S21. (a) Top view and (b) side view of the DFT-optimized structures of Ni-BDC. (c) Top view and (d) side view of the DFT-optimized structures of NiMn-BDC.

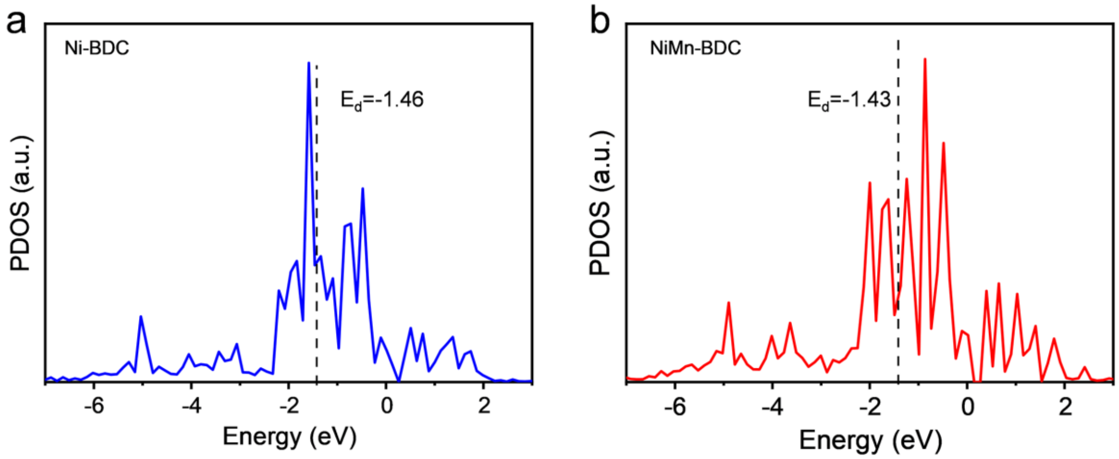

Figure S22. The calculated partial density of states (PDOS) of *d* orbitals for (a) Ni-BDC and (b) NiMn-BDC.

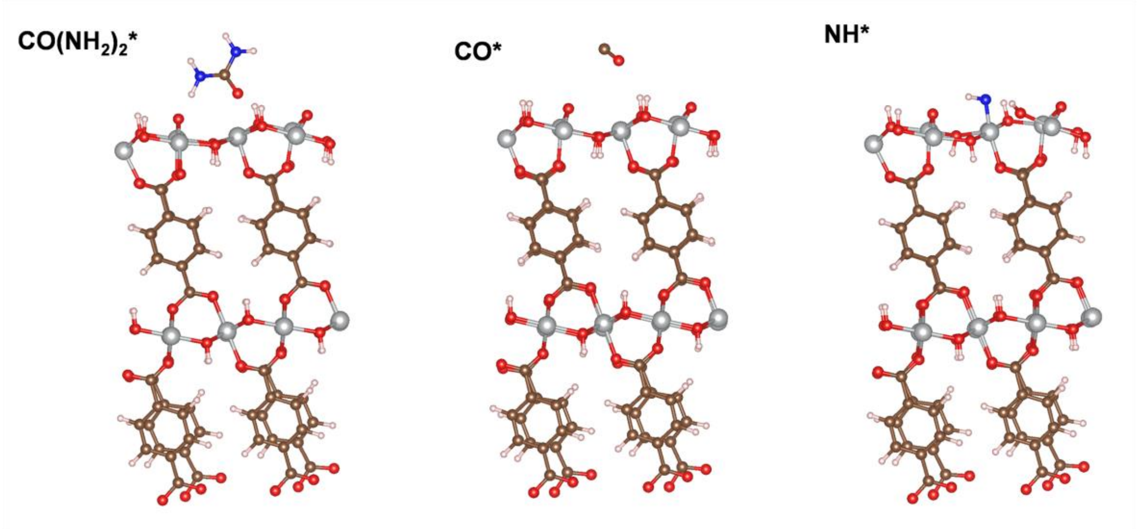

Figure S23. The corresponding structural evolution of UOR reaction intermediates adsorbed at the Ni in Ni-BDC.

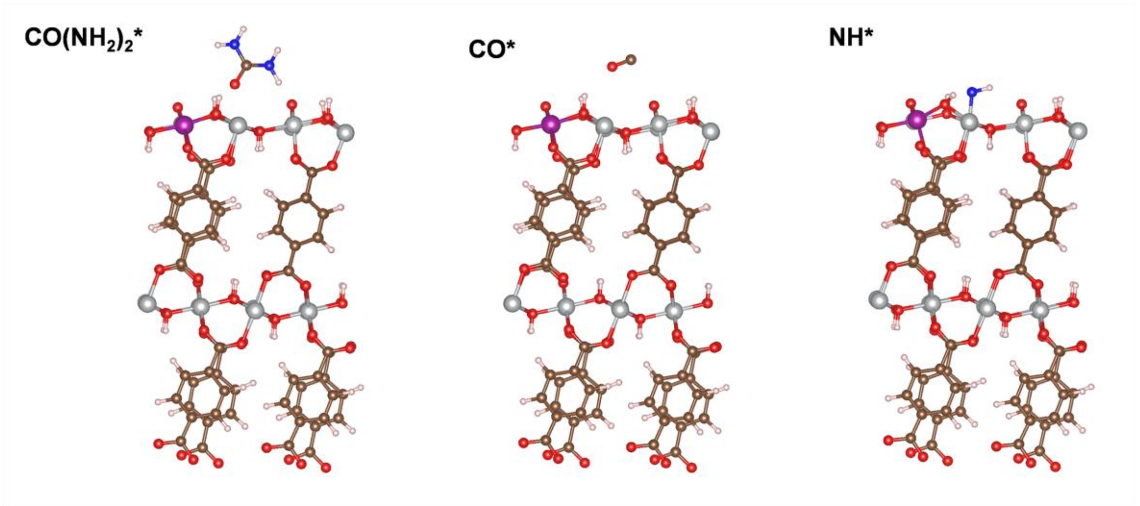

Figure S24. The corresponding structural evolution of UOR reaction intermediates adsorbed at the Ni in NiMn-BDC.

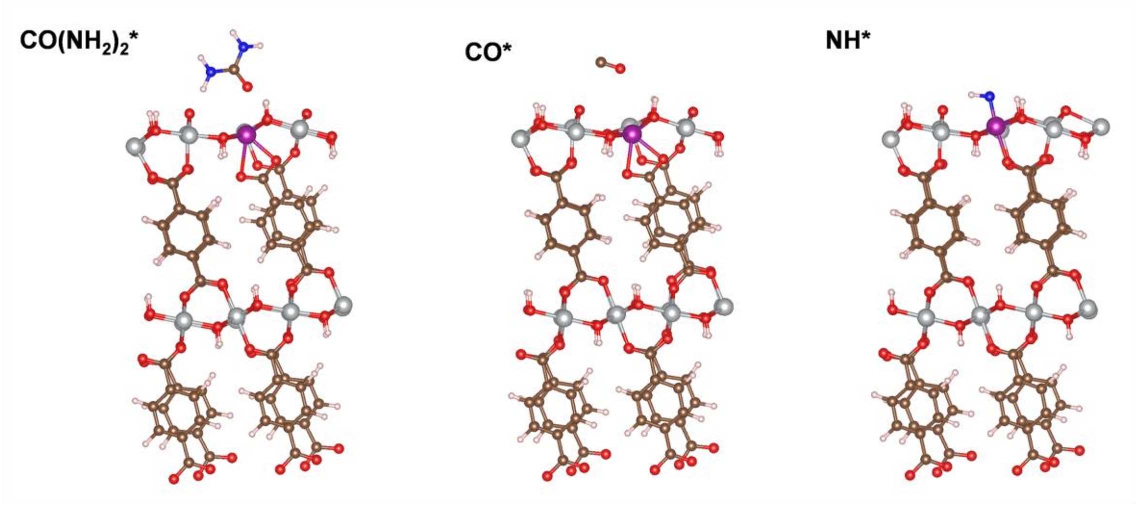

Figure S25. The corresponding structural evolution of UOR reaction intermediates adsorbed at the Mn in NiMn-BDC.

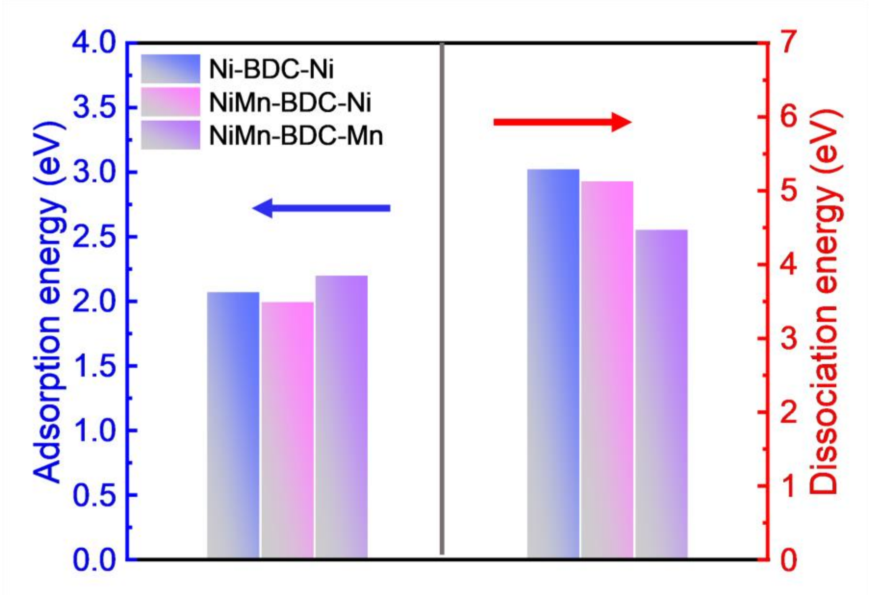

Figure S26. Adsorption energy of urea (left) and adsorption energy change from $CO(NH_2)_2$* to NH* and CO* intermediates (dissociation energy of $CO(NH_2)_2$*) (right) at the Ni in NiMn-BDC, the Ni in Ni-BDC and the Mn in NiMn-BDC.

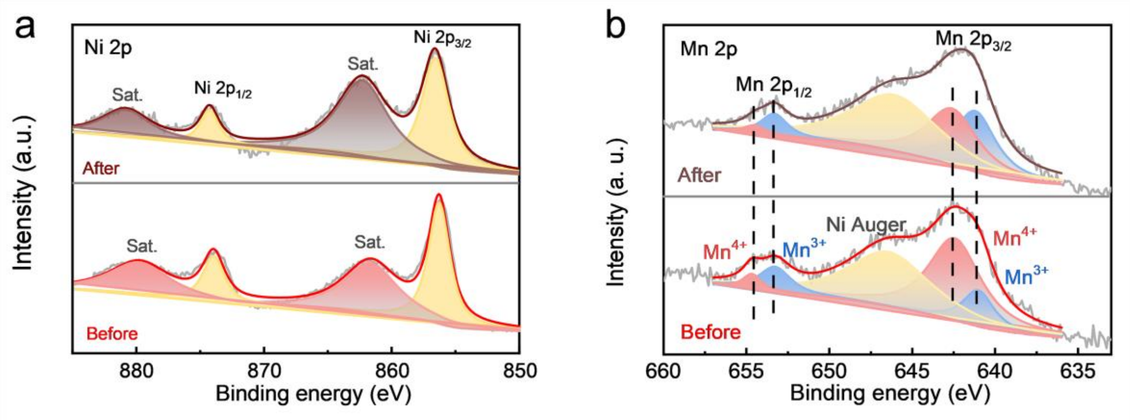

Figure S27. XPS analysis of $NiMn_{0.14}$-BDC after UOR stability test.

19.

Movie S1. Electrochemical-driven urea degradation. A two-electrode full-cell was constructed for evaluation of the electrochemically driven urea degradation performance, in which $NiMn_{0.14}$-BDC/NF works as both the cathode and the anode. From the movie, it can be found that gas was continuously bubbling on the cathode and anode, signifying that the urea is successfully splited into N_2 and CO_2 during the UOR process.

Table S1. Structural parameters of Ni-BDC and $NiMn_{0.14}$-BDC. Fourier-transforms (FT) are performed in the *K*-range between 0 $Å^{-1}$ and 12 $Å^{-1}$.

Samples	Path	CN	R(Å)	DW($Å^2$)
Ni-BDC	Ni-O	5.5(3)	2.11(1)	0.0033(5)
$NiMn_{0.14}$-BDC	Ni-O	5.7(5)	2.10(1)	0.0053(9)
$NiMn_{0.14}$-BDC	Mn-O	5.8(6)	2.10(3)	0.0052(3)

R(Å): Apparent distance
CN: Coordination number
DW: Debye-Waller parameter

Table S2. The fitted EIS resistance of $NiMn_x$-BDC.

	**R_s (Ω)**	**R_p (Ω)**	**R_{ct} (Ω)**
Ni-BDC	5.75	2.94	96.51
$NiMn_{0.12}$-BDC	5.48	2.20	14.20
$NiMn_{0.13}$-BDC	5.25	1.87	9.20
$NiMn_{0.14}$-BDC	5.35	0.31	5.84
$NiMn_{0.16}$-BDC	5.47	1.72	7.99
$NiMn_{0.19}$-BDC	5.29	2.23	13.63

R_s: The system resistance
R_p: The electrode porosity resistance
R_{ct}: The charge transfer resistance

Table S3. UOR performance of $NiMn_{0.14}$-BDC and the recently reported Ni-based and Mn-based electrocatalysts.

	Electrolyte	10 (mA cm^{-2})	18 (mA cm^{-2})	50 (mA cm^{-2})	60 (mA cm^{-2})	100 (mA cm^{-2})	Ref.
$NiMn_{0.14}$-BDC	1.0 M KOH +0.33 M urea	1.32	1.33	1.35	1.36	1.37	This work
RuO_2	1.0 M KOH +0.5 M urea	1.35	–	–	–	–	[2]
Rh-Ni	1.0 M KOH +0.5 M urea	1.45	–	–	–	–	[2]
Ni-MOF-t	1.0 M KOH +0.5 M urea	1.38	–	–	–	–	[3]
Ni-MOF	1.0 M KOH +0.33 M urea	1.36	–	–	–	–	[4]
M-$Ni(OH)_2$	1.0 M KOH +0.33 M urea	–	1.48	–	–	–	[5]
Ni-WOx	1.0 M KOH +0.33 M urea	–	–	–	–	1.40	[6]
Ni-Mo nanotube	1.0 M KOH +0.5 M urea	1.36	–	–	–	–	[7]
r-$NiMoO_4$	1.0 M KOH +0.5 M urea	–	–	–	1.42	–	[8]
NF/NiMoO-Ar	1.0 M KOH +0.33 M urea	–	–	–	–	1.42	[9]
Ni_2P NF/CC	1.0 M KOH +0.33 M urea	–	–	–	1.46	1.52	[10]
Ni-CoP/HPFs	1.0 M KOH +0.5 M urea	–	–	–	1.62	–	[11]
NiClO-D	1.0 M KOH +0.5 M urea	–	–	1.39	–	–	[12]
NF-G-Mn	1.0 M KOH +0.33 M urea	1.33	–	–	–	–	[13]
$MnO_2/MnCo_2O_4$/Ni	1.0 M KOH +0.5 M urea	1.33	–	–	–	–	[14]
CoMn-LDH	1.0 M KOH +0.33 M urea	–	–	1.38	–	–	[15]
Mn-Ni_3S_2/NF	1.0 M KOH +0.5 M urea	–	–	–	–	1.39	[16]

Table S4. The absorbance value of urea solutions.

Time (min)	0.0033 M	0.033 M	0.33 M
0	1.013	0.963	1.085
20	0.591	0.623	0.960
40	0.285	0.473	0.804
60	0.153	0.245	0.721
80	0.109	0.196	0.564
100	0.086	0.134	0.468
120	0.073	0.106	0.343
140	0.063	0.091	0.284
160	0.059	0.079	0.232
180	0.056	0.070	0.186

Table S5. The absorbance value of $NiMn_{0.14}$-BDC after different cycles.

Time (min)	The first cycle	The second cycles	The third cycles
0	1.096	1.068	1.080
20	0.977	0.995	0.995
40	0.817	0.831	0.819
60	0.757	0.741	0.737
80	0.564	0.532	0.552
100	0.448	0.469	0.489
120	0.377	0.404	0.426
140	0.309	0.364	0.327
160	0.234	0.286	0.272
180	0.196	0.217	0.223

References for Supporting Information

[1] F. L. Li, P. Wang, X. Huang et al., "Large-Scale, Bottom-Up Synthesis of Binary Metal-Organic Framework Nanosheets for Efficient Water Oxidation," *Angewandte Chemie International Edition*, vol. 58, no. 21, pp. 7051-7056, 2019.
[2] A. Kumar, X. Liu, J. Lee *et al.*, "Discovering ultrahigh loading of single-metal-atoms via surface tensile-strain for unprecedented urea electrolysis," *Energy & Environmental Science,* vol. 14, no. 12, pp. 6494-6505, 2021.
[3] S. Zheng, Y. Zheng, H. Xue *et al.*, "Ultrathin nickel terephthalate nanosheet three-dimensional aggregates with disordered layers for highly efficient overall urea electrolysis," *Chemical Engineering Journal,* vol. 395, article 125166, 2020.
[4] D. Zhu, C. Guo, J. Liu *et al.*, "Two-dimensional metal-organic frameworks with high oxidation states for efficient electrocatalytic urea oxidation," *Chemical Communications,* vol. 53, no. 79, pp. 10906-10909, 2017.
[5] X. Zhu, X. Dou, J. Dai *et al.*, "Metallic Nickel Hydroxide Nanosheets Give Superior Electrocatalytic Oxidation of Urea for Fuel Cells," *Angewandte Chemie International Edition,* vol. 55, no. 40, pp. 12465-12469, 2016.
[6] L. Wang, Y. Zhu, Y. Wen *et al.*, "Regulating the Local Charge Distribution of Ni Active Sites for the Urea Oxidation Reaction," *Angewandte Chemie International Edition,* vol. 60, no. 19, pp. 10577-10582, 2021.
[7] J.-Y. Zhang, T. He, M. Wang *et al.*, "Energy-saving hydrogen production coupling urea oxidation over a bifunctional nickel-molybdenum nanotube array," *Nano Energy,* vol. 60, pp. 894-902, 2019.
[8] Y. Tong, P. Chen, M. Zhang *et al.*, "Oxygen Vacancies Confined in Nickel Molybdenum Oxide Porous Nanosheets for Promoted Electrocatalytic Urea Oxidation," *ACS Catalysis,* vol. 8, no. 1, pp. 1-7, 2017.
[9] Z.-Y. Yu, C.-C. Lang, M.-R. Gao *et al.*, "Ni–Mo–O nanorod-derived composite catalysts for efficient alkaline water-to-hydrogen conversion via urea electrolysis," *Energy & Environmental Science,* vol. 11, no. 7, pp. 1890-1897, 2018.
[10] D. Liu, T. Liu, L. Zhang *et al.*, "High-performance urea electrolysis towards less energy-intensive electrochemical hydrogen production using a bifunctional catalyst electrode," *Journal of Materials Chemistry A,* vol. 5, no. 7, pp. 3208-3213, 2017.
[11] Y. Pan, K. Sun, Y. Lin *et al.*, "Electronic structure and d-band center control engineering over M-doped CoP (M = Ni, Mn, Fe) hollow polyhedron frames for boosting hydrogen production," *Nano Energy,* vol. 56, pp. 411-419, 2019.
[12] L. Zhang, L. Wang, H. Lin *et al.*, "A Lattice-Oxygen-Involved Reaction Pathway to Boost Urea Oxidation," *Angewandte Chemie International Edition,* vol. 58, no. 47, pp. 16820-16825, 2019.
[13] S. Chen, J. Duan, A. Vasileff, *et al.*, "Size fractionation of two-dimensional sub-nanometer thin manganese dioxide crystals towards superior urea electrocatalytic conversion," *Angewandte Chemie International Edition.* vol.55, no. 11, pp. 3804-3808, 2016.
[14] C. Xiao, S. Li, X. Zhang *et al.*, "$MnO_2/MnCo_2O_4$/Ni heterostructure with quadruple hierarchy: a bifunctional electrode architecture for overall urea oxidation," *Journal of Materials Chemistry A,* vol. 5, no. 17, pp. 7825-7832, 2017.
[15] Z. Wang, Y. Hu, W. Liu *et al.*, "Manganese-Modulated Cobalt-Based Layered Double Hydroxide Grown on Nickel Foam with 1D-2D-3D Heterostructure for Highly Efficient Oxygen Evolution Reaction and Urea Oxidation Reaction," *Chemistry,* vol. 26, no. 42, pp. 9382-9388, 2020.
[16] H. Yang, M. Yuan, Z. Sun *et al.*, "In Situ Construction of a Mn^{2+}-Doped Ni_3S_2

Electrode with Highly Enhanced Urea Oxidation Reaction Performance," *ACS Sustainable Chemistry & Engineering,* vol. 8, no. 22, pp. 8348-8355, 2020.
